# Mesenchymal stromal cells ameliorate diabetes‐induced muscle atrophy through exosomes by enhancing AMPK/ULK1‐mediated autophagy

**DOI:** 10.1002/jcsm.13177

**Published:** 2023-01-27

**Authors:** Jia Song, Jidong Liu, Chen Cui, Huiqing Hu, Nan Zang, Mengmeng Yang, Jingwen Yang, Ying Zou, Jinquan Li, Lingshu Wang, Qin He, Xinghong Guo, Ruxing Zhao, Fei Yan, Fuqiang Liu, Xinguo Hou, Zheng Sun, Li Chen

**Affiliations:** ^1^ Department of Endocrinology Qilu Hospital of Shandong University Jinan Shandong China; ^2^ Institute of Endocrine and Metabolic Diseases of Shandong University Jinan Shandong China; ^3^ Key Laboratory of Endocrine and Metabolic Diseases, Shandong Province Medicine & Health Jinan Shandong China; ^4^ Jinan Clinical Research Center for Endocrine and Metabolic Disease Jinan Shandong China

**Keywords:** Muscle atrophy, hucMSCs, Exosome, AMPK/ULK1, Autophagy

## Abstract

**Background:**

Diabetes and obesity are associated with muscle atrophy that reduces life quality and lacks effective treatment. Mesenchymal stromal cell (MSC)‐based therapy can ameliorate high fat‐diet (HFD) and immobilization (IM)‐induced muscle atrophy in mice. However, the effect of MSCs on muscle atrophy in type 2 diabetes mellitus (T2DM) and the potential mechanism is unclear. Here, we evaluated the efficacy and explored molecular mechanisms of human umbilical cord MSCs (hucMSCs) and hucMSC‐derived exosomes (MSC‐EXO) on diabetes‐ and obesity‐induced muscle atrophy.

**Methods:**

Diabetic db/db mice, mice fed with high‐fat diet (HFD), mice with hindlimb immobilization (IM), and C2C12 myotubes were used to explore the effect of hucMSCs or MSC‐EXO in alleviating muscle atrophy. Grip strength test and treadmill running were used to measure skeletal muscle strength and performance. Body composition, muscle weight, and muscle fibre cross‐sectional area (CSA) was used to evaluate muscle mass. RNA‐seq analysis of tibialis anterior (TA) muscle and Western blot analysis of muscle atrophy signalling, including MuRF1 and Atrogin 1, were performed to investigate the underlying mechanisms.

**Results:**

hucMSCs increased grip strength (*P* = 0.0256 in db/db mice, *P* = 0.012 in HFD mice, *P* = 0.0097 in IM mice), running endurance (*P* = 0.0154 in HFD mice, *P* = 0.0006 in IM mice), and muscle mass (*P* = 0.0004 in db/db mice, *P* = 0.0076 in HFD mice, *P* = 0.0144 in IM mice) in all models tested, with elevated CSA of muscle fibres (*P* < 0.0001 in db/db mice and HFD mice, *P* = 0.0088 in IM mice) and reduced Atrogin1 (*P* = 0.0459 in db/db mice, *P* = 0.0088 in HFD mice, *P* = 0.0016 in IM mice) and MuRF1 expression (*P* = 0.0004 in db/db mice, *P* = 0.0077 in HFD mice, *P* = 0.0451 in IM mice). MSC‐EXO replicated all these hucMSC‐mediated changes (*P* = 0.0103 for grip strength, *P* = 0.013 for muscle mass, *P* < 0.0001 for CSA of muscle fibres, *P* = 0.0171 for Atrogin1 expression, and *P* = 0.006 for MuRF1 expression). RNA‐seq revealed that hucMSCs activated the AMPK/ULK1 signalling and enhanced autophagy. Knockdown of AMPK or inhibition of autophagy with 3‐methyladenine (3‐MA) diminished the beneficial anti‐atrophy effects of hucMSCs or MSC‐EXO.

**Conclusions:**

Our results suggest that human umbilical cord mesenchymal stromal cells mitigate diabetes‐ and obesity‐induced muscle atrophy via enhancing AMPK/ULK1‐mediated autophagy through exosomes, with implications of applying hucMSCs or hucMSC‐derived exosomes to treat muscle atrophy.

## 1 Introduction

Loss of muscle mass and function is associated with aging, muscle disuse/immobility, and chronic diseases such as type 2 diabetes mellitus (T2DM) and obesity.^1^, ^2^ Patients with T2DM exhibit mild muscle atrophy in middle age^3^ and become more severe with aging and diabetic neuropathy.^4^, ^5^ The causal role of obesity, diabetes, and immobility in muscle atrophy has been demonstrated in animal models. Obese db/db mice and mice fed with a high‐fat diet (HFD) develop the typical features of muscle wasting, including weakness, the loss of muscle mass, and decreased fibre diameter.^6^, ^7^ Muscle atrophy also occurs rapidly in rodents in response to disuse through joint immobilization, hind limb unloading, or spinal cord injury.^8^

Muscle atrophy results from a negative balance between the rate of contractile protein synthesis and degradation. Insulin resistance in T2DM and obesity is a key contributor to muscle atrophy.^9^ Impairment of the insulin signalling pathway is not only implicated in decreasing protein synthesis,^10^ but also associated with an increased rate of muscle protein degradation.^11^ The ubiquitin‐proteasome and autophagy‐lysosome systems are crucial to muscle protein degradation.^12^ E3 ubiquitin ligases muscle RING Finger 1 (MuRF1) and muscle atrophy F‐box (MAFbx)/Atrogin 1 are key components of the ubiquitin‐proteasome system that contribute to muscle atrophy.^13^ These E3 ubiquitin ligases were shown to be upregulated in diabetes, obesity, and muscle disuse.^7^, ^13^, ^14^

In addition to the ubiquitin‐proteasome system, autophagy is also crucial for the maintenance of muscle integrity and function. Inhibition of autophagy has been reported to aggravate muscle atrophy.^15^ Aging‐related muscle atrophy could be caused by inhibition of autophagy characterized by accumulation of LC3 and p62.^16^ Skeletal muscle fibres from older, overweight sarcopenic individuals also present an impaired autophagic flux.^17^ AMPK is a positive regulator of autophagy through phosphorylation of ULK1 at several primary sites, including Ser555, Ser317, and Ser777. AMPK muscle‐specific deletion in aged mice impairs muscle autophagy.^18^ Deletion of AMPK from skeletal muscle dramatically reduces the exercise capacity of mice,^19^ whereas AMPK activation ameliorates muscle dystrophy.^20^ AMPK is also an important intracellular energy sensor regulating metabolic homeostasis,^21^ which is crucial for maintaining skeletal muscle function.

Mesenchymal stromal cell (MSC)‐based therapy is promising in alleviating diabetes and its complications.^22^, ^23^ MSCs can be obtained from a variety of sources, such as embryos, bone marrow, adipose tissue and umbilical cord.^24^ It has been reported that bone marrow‐mesenchymal stromal cells (BM‐MSCs), adipose‐derived stromal cells (AD‐MSCs) and embryonic stromal cells (ESCs) are effective in preventing muscle atrophy.^25^ ^,S1‐S3^ In comparison with other MSCs, human umbilical cord‐mesenchymal stromal cells (hucMSCs) are easier to obtain, have higher proliferative potential and lower immunogenicity, which makes hucMSCs an ideal choice for regenerative therapy.^26^ However, the effect of hucMSCs on muscle atrophy in T2DM pre‐clinical models is unclear. The mechanism for how hucMSCs alleviate muscle atrophy is not completely understood. It was speculated that MSCs might directly generate new myofibers. However, previous tracing studies did not observe MSCs incorporation into the muscle tissues in HFD or IM mouse models.^25^, ^27^ We suspect that indirect events might underlie MSC‐dependent anti‐atrophy effects. Accumulating studies suggest that the therapeutic effects MSCs in other diseases are related to its paracrine action.^28^, ^29^ Exosomes secreted by MSCs contain multiple bioactive molecules, which could mediate the therapeutic effects of MSCs.^30^ ^,S4^ Here, we characterize the effects of hucMSCs or hucMSC‐derived exosomes on diabetes‐ and obesity‐associated muscle atrophy in multiple mouse models *in vivo* and fully‐differentiated C2C12 myotubes *in vitro*.

## 2 Methods

### 2.1 Human MSCs and exosomes

Fresh human umbilical cords were obtained from healthy newborns using the protocol approved by the Ethics Committee of Shandong University Qilu Hospital. All participants provided informed consent. After washing the umbilical cords and removing blood vessels, we cut Wharton's jelly into small pieces and spread them in cell culture flasks with α‐MEM medium (Gibco, USA) containing 10% fetal bovine serum (FBS; Gibco) and 100 U/mL penicillin and 100 μg/mL streptomycin (Gibco). The culture medium was changed every 3 days until the cells reached 80% confluency. The third to the fifth passage of cells were used for flow cytometry analysis, differentiation induction, administration, and Transwell™ co‐culturing. After culture in the exosome‐free medium for 48 h, hucMSC‐conditioned medium was collected and centrifuged to remove cells and cell debris, followed by filtration through a 0.22‐μm filter. Subsequently, the medium was ultracentrifuged at 120 000× *g* for 70 min at 4°C. The exosomes were collected as the pellet. The sizes of exosomes were quantified by ZetaVIEW S/N 17‐310 (PARTICLE METRIX, Germany). Morphologies of exosomes were detected by transmission electron microscopy (TEM; FEI, Tecnai G2 Spirit BioTwin, USA). The expressions of CD9, CD63, and CD81 were assessed by Western blot.

### 2.2 Animals

Four‐week‐old male db/db mice^S5^ were purchased from Changzhou Kavins Laboratory Animal Co., Ltd. (Changzhou, China) and were fed a normal chow diet. The mice were housed in a 12‐h light/dark cycle at a temperature (22–25°C)‐ and humidity (55% ± 5%)‐controlled environment. T2DM was identified as fasting glucose ≥16.7 mmol/L twice in succession. Six‐week‐old male C57BL/6 J mice were purchased from the Model Animal Research Center of Shandong University (Jinan, China). The obesity‐related muscle atrophy mice model was established by ad libitum feeding HFD (Jiangsu Xietong Pharmaceutical Bio‐Engineering Co., Ltd., Nanjing, China)^25^ for 30 weeks started from 8 weeks old. Then, hucMSCs (1 × 10^6^ cells per mouse) were suspended in PBS and injected into the above two kinds of mice via the tail vein every 7 days for eight cycles. For hucMSC‐derived exosomes (MSC‐EXO) injection, 200 μg MSC‐EXO was injected through the tail vein every 3 days for 8 weeks. For the disuse muscle atrophy model, 6‐week‐old male C57BL/6 J mice were immobilized for 2 weeks using soft plastic‐coated wire ties, which imitated the immobilization method with Velcro tape or plaster casts as previously described.^S1,S6^ Briefly, soft plastic‐coated wire ties were cut to 20–25 cm lengths and used to immobilize the left hind limb from the foot to the thigh, resulting in IM of the knee in the extension position and the ankle in the plantar flexion position. Then, hucMSCs (1 × 10^6^ cells per mouse) in PBS were injected into tibialis anterior and soleus muscles in fixed limbs every 7 days for four cycles (IM + hucMSCs treatment group). The same volume of PBS without hucMSCs was given to controls (IM + PBS treatment group). All animal experiments were approved by the Animal Ethics Committee of Shandong University.

### 2.3 Metabolic testing and *in vivo* muscle performance analysis

Body composition was measured using dual‐energy X‐ray absorptiometry (DXA, Ge Lunar Prodigy, USA). The lean mass proportion was expressed as the percentage of lean mass composition to body composition. The intraperitoneal glucose tolerance test (IPGTT) and intraperitoneal insulin tolerance test (IPITT) as previously described.^31^ The exhaustive running distance was measured using a treadmill (shanghai xinrun information technology Co., Ltd., China). After 2 days of acclimation, the exercise capacity of mice was determined by measuring their ability to run until exhaustion. Mice were first subjected to running at 8 m/min for 20 min. Thereafter, the treadmill speed was increased by 0.2 m/min. Exhaustion was defined as hindlimbs remaining on the electric grid for more than 10 s. Grip strength was determined by an electronic dynamometer (shanghai xinrun information technology Co., Ltd.). We trained the mice to grasp the horizontal grid connected to the dynamometer with four limbs and gently pulled them backward in a horizontal direction parallel to the dynamometer. The force applied to the grid each time before the mouse lost its grip was recorded. The grip strength test of each mouse was performed thrice, and the three measured values were recorded and averaged.

### 2.4 Histology and immunostaining

After the mice were anaesthetised, bilateral tibialis anterior (TA) muscles were dissected and weighed. One muscle was frozen at −80°C after liquid nitrogen treatment, and the other was fixed in 4% paraformaldehyde for haematoxylin–eosin (H&E) staining, Masson staining, Sirius Red staining and immunostaining. The muscle weight was normalized to body weight. The TA muscle samples fixed in 4% paraformaldehyde were embedded in paraffin and were sliced at the maximum cross‐section with a thickness of 5 μm. After dewaxed, slides were used for H&E staining, Masson staining and Sirius Red staining following the standard procedures. For immunohistochemical staining, after slides were dewaxed, antigen retrieval was performed using antigen unmasking buffer. After treating with 3% hydrogen peroxide to inactivate endogenous peroxidases for 15 min, and blocking at room temperature for 30 min in a protein‐blocking solution (10% normal goat serum), the slides were incubated with anti‐Fast Myosin Skeletal Heavy Chain (Abcam, USA, Cat. No. ab91506, 1:1000) and anti‐Slow Myosin Skeletal Heavy Chain antibodies (Abcam, Cat. No. ab234431, 1:1000) overnight at 4°C. The slides were then incubated with secondary antibody at room temperature for 60 min and then stained with 3,3′‐diaminobenzidine (DAB) solution. Images were acquired using a microscope (Olympus BX53, Japan), and the cross‐sectional area (CSA) of muscle fibres was calculated using Image‐Pro Plus software. For immunofluorescence staining, after slides were dewaxed, antigen retrieval was performed using antigen unmasking buffer. After blocking at room temperature for 30 min in a protein‐blocking solution (10% normal goat serum), the slides were incubated with anti‐rabbit LC3 antibody (Abcam, Cat. No. ab192890, 1:1000) overnight at 4°C. The slides were then incubated with goat anti‐rabbit FITC (Zhongshan, Beijing, China, Cat: ZF‐0311, 1:200) at room temperature for 60 min and then stained with DAPI for 5 min. Fluorescence images were observed and captured using a fluorescence microscope (Olympus BX53, Japan). The proportion of LC3‐positive muscle fibres was analysed and calculated by ImageJ software.

### 2.5 Exosomal tracing

For exosomal tracing *in vivo*, MSC‐EXO was labelled with Cy7 (MedChemExpress, USA) and injected into mice via tail vein. Mice were imaged using an *in vivo* imaging system (IVIS) (Xenogen Corp., Alameda, CA, USA) at 12 h after injection. For exosomal tracing *in vitro*, MSC‐EXO were labelled using the PKH67 Green Fluorescent Cell Linker Kit (PKH67, Sigma‐Aldrich) according to the manufacturer's instructions, followed by incubation with fully differentiated myotubes for 24 h. Fluorescence signals were detected and captured by a fluorescence microscope (Olympus BX53, Japan).

### 2.6 Cell lines and RNAi

Human embryo lung fibroblasts (HELFs) were obtained from the China Cell Culture Center (Shanghai, China) and cultured in Dulbecco's modified Eagle's medium (DMEM)/high‐glucose medium (Gibco, USA) supplemented with 10% FBS, 100 U/mL penicillin and 100 μg/mL streptomycin at 37°C and in a 5% CO_2_ incubator. Mouse C2C12 myoblasts were obtained from China Infrastructure of Cell Line Resource (Beijing, China) and cultured in DMEM/high‐glucose medium (Gibco, USA) supplemented with 10% FBS and antibiotics. After reaching 80%–90% confluency, the medium was replaced with a differentiation medium consisting of DMEM plus 2% heat‐inactivated horse serum and antibiotics for 4 days. The medium was refreshed every 2 days. The fully differentiated myotubes were stimulated with palmitate (PA, 0.6 mM) for 24 h, followed by treatment with hucMSCs or HELFs via a Transwell™ system (Catalogue No. 3450; Corning, USA) and MSC‐EXO (25 μg/mL) for 24 h. To further explore the mechanism of hucMSCs and MSC‐EXO, myotubes were pre‐treated with small interfering RNA (siRNA) or autophagy inhibitor 3‐methyladenine (3‐MA, 2 mM, Sigma‐Aldrich, USA) before treatment with hucMSCs and MSC‐EXO. siRNA oligonucleotides were synthesized by GenePharma Co., Ltd (Shanghai, China). The sequences of negative control (NC) siRNA were as follows: sense 5′‐UUCUCCGAACGUGUCACGUTT‐3′ and antisense 5′‐ACGUGACACGUUCGGAGAATT‐3′. The sequences for the AMPKα2 siRNA were as follows: sense 5′‐CCCAGAUGAACGCUAAGAUTT‐3′ and antisense 5′‐AUCUUAGCGUUCAUCUGGGTT‐3′. C2C12 myotubes were transfected with siRNA for AMPKα2 using Lipofectamine 2000 transfection reagent (Invitrogen, USA) following the manufacturer's instructions. Briefly, the C2C12 myoblasts were plated in 6‐well plates and were fully differentiated. The medium was then removed and replaced by Opti‐MEM I reduced serum medium (Gibco) mixed with siRNA for AMPKα2 (125 nM) for 6 h. Subsequently, the culture medium was replaced by the differentiation medium, and myotubes were treated with hucMSCs or MSC‐EXO. After 24 h, myotubes were collected for the next experiments.

### 2.7 Western blot

TA muscle samples of mice were lysed in radioimmunoprecipitation assay (RIPA) lysis buffer (P0013B, Beyotime, Shanghai, China), and protein concentration was detected with the bicinchoninic acid (BCA) method (Beyotime, China). Subsequently, proteins were separated and transferred onto polyvinylidene difluoride (PVDF) membranes (IPVH00010 0.45 μm, Millipore, USA), and the membranes were blocked with 5% skim milk for 1 h at room temperature and incubated in specific primary antibodies at 4°C overnight. After incubation with horseradish peroxidase‐conjugated secondary antibodies for 1 h at room temperature, the proteins were visualized using enhanced chemiluminescence. The primary antibodies were as follows: Atrogin1 (Proteintech, China, Cat. No. 67172‐1‐Ig, 1:5000), MuRF1 (Proteintech, Cat. No. 55456‐1‐AP, 1:1000), AMP‐activated protein kinase (AMPK, USA, CST, Cat. No. 5831S, 1:1000), phosphorylated‐(p‐)AMPK (Thr172) (CST, Cat. No. 2535S, 1:1000), ULK1 (Bioss, China, Cat. No. bs‐3602R, 1:500), phosphorylated‐(p‐)ULK1 (Immunoway, USA, Cat. No. YP1542, 1:1000), LC3 (Proteintech, Cat. No. 14600‐1‐AP, 1:1000), p62 (Abcam, USA, Cat. No. ab91526, 1:1000), GADPH (Boster, China, Cat. No. BA2913, 1:500).

### 2.8 RNA‐seq and real‐time quantitative PCR analysis

Total RNA was isolated using Trizol reagent (Invitrogen, USA) following the manufacturer's procedure. The library construction and sequencing were performed at Shenzhen BGI Genomics Co., Ltd. STAR (v.2.5) was used to index the mouse reference genome (mm10) and align the resulting fastq files. DESeq2 (v1.28.1) was applied to perform the differential expression analysis. KEGG pathway enrichment was implemented by the ClusterProfiler (v3.10.1). Reverse transcription was performed with 1 μg RNA using the Prime Script RT Reagent Kit (Cat. No. RR047A; Takara, Japan). Primers were chemically synthesized by GenePharma Co., Ltd. (Shanghai, China). The primer sequences were as follows: *Fbxo32*, sense 5′‐GGGGTCACCCTGCAGCTTTGC‐3′ and antisense 5′‐GGGGAAAGTGAGACGGAGCAGC‐3′; *Trim63*, sense 5′‐ATGGACCGGCACGGGGTGTA‐3′ and antisense 5′‐GCACATCGGGTGGCTGCCTT‐3′; *Ulk1*, sense 5′‐AAGTTCGAGTTCTCTCGCAAG‐3′ and antisense 5′‐CGATGTTTTCGTGCTTTAGTTCC‐3′; *Map 1lc3b*, sense 5′‐TTATAGAGCGATACAAGGGGGAG‐3′ and antisense 5′‐CGCCGTCTGATTATCTTGATGAG‐3′; *Sqstm1*, sense 5′‐AGGATGGGGACTTGGTTGC‐3′ and antisense 5′‐TCACAGATCACATTGGGGTGC‐3′; *Gapdh*, sense 5′‐AAGGGCTCATGACCACAGTC‐3′ and antisense 5′‐CAGGGATGATGTTCTGGGCA‐3′. Real‐time PCR was conducted using the SYBR Green PCR Kit (Cat. No. RR420A; Takara), gene expression changes were determined with the comparative CT (2^−ΔΔCt^) method, and quantification was achieved by normalization using *Gapdh* as control.

### 2.9 Statistical analysis

All of the data were presented as the mean ± SEM. Differences between the groups were evaluated using unpaired Student's *t*‐test or one‐way ANOVA followed by Tukey's test via GraphPad Prism 8 software. *P* < 0.05 was considered to be statistically significant.

## 3 Results

### 3.1 hucMSCs alleviate diabetes‐induced muscle atrophy

**Figure 1:**
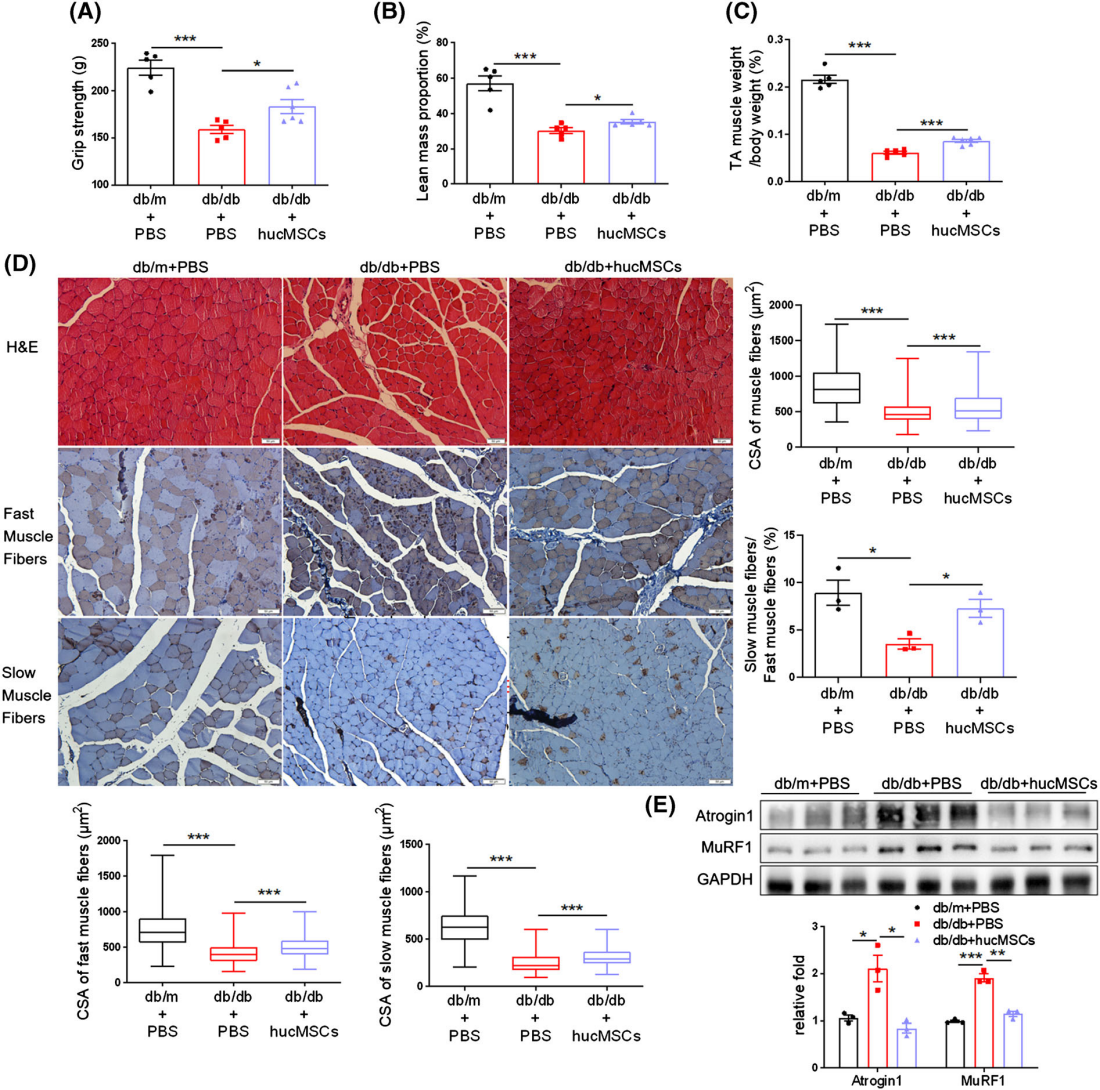
hucMSCs alleviate diabetes‐induced muscle atrophy. (A) Grip strength (*n* = 5–6 mice). (B) Lean mass proportion detected by dual‐energy X‐ray absorptiometry (DXA) (*n* = 5–6 mice). (C) Percentage of tibialis anterior (TA) muscle weight in body weight (*n* = 5–6 mice). (D) Haematoxylin–eosin (H&E) staining and immunohistochemical staining of fast and slow myosin heavy chain of TA muscles (scale bar, 50 μm). And cross‐sectional area (CSA) of muscle fibres, fast and slow muscle fibres and the ratio of slow muscle fibres to fast muscle fibres (*n* = 3–5 mice). (E) Western blot analysis of Atrogin 1 and MuRF1 in TA muscles (*n* = 3 mice). Quantification of bands was performed using ImageJ software. Data are mean ± SEM. (*P < 0.05, **P < 0.01, ***P < 0.001 by two‐sided unpaired Student's *t*‐test).

We used the db/db mice^S5^ to investigate whether hucMSCs alleviate muscle atrophy in T2DM. Primary hucMSCs were isolated and identified as previously described.^32^ Flow cytometry analysis showed that hucMSCs were positive for stem cell markers CD105 and CD73, and negative for CD34 and HLA‐DR (*Figure* S1A). Oil Red O staining and Alizarin Red S staining indicated that hucMSCs can be differentiated into adipocytes and osteoblasts, respectively (*Figure* S1B,C). We administered hucMSCs (1 × 10^6^ cells per mouse suspended in 200 μL PBS) into db/db mice from 5 weeks old via the tail vein every 7 days for a total of 8 injections; 200 μL per mouse PBS injection served as the control. IPGTT and IPITT at 1 week after the 8 th injection showed that hucMSCs improved glucose and insulin tolerance (*Figure* *S2* *A,B*). The grip strength test indicated decreased muscle strength in db/db mice compared with db/m mice, which was improved by hucMSC injection (*Figure* 1A). hucMSC injection did not affect body weight and lean mass (*Figure* *S2* *C‐D*), but elevated lean mass proportion (*Figure* 1B) and increased the tibialis anterior (TA) muscle mass (*Figures* 1A and S2E). The cross‐sectional area (CSA) of muscle fibres, including fast and slow muscle fibres in db/db mice, was remarkably smaller than that in db/m mice, and hucMSC treatment alleviated diabetes‐induced reduction of CSA of muscle fibres, increased the percentage of slow in fast muscle fibres and promoted muscle fibre formation (*Figures* 1D and S2F). hucMSC injection also reduced E3‐ubiquitin ligases Atrogin 1 and MuRF1 expression (*Figure* 1E). In addition, muscle fibrosis in db/db mice was also ameliorated by hucMSCs (*Figure* *S2* *G*). These results suggest that hucMSC injection alleviates diabetes‐induced muscle atrophy in db/db mice without affecting body weight.

### 3.2 hucMSCs alleviate obesity‐induced muscle atrophy

**Figure 2:**
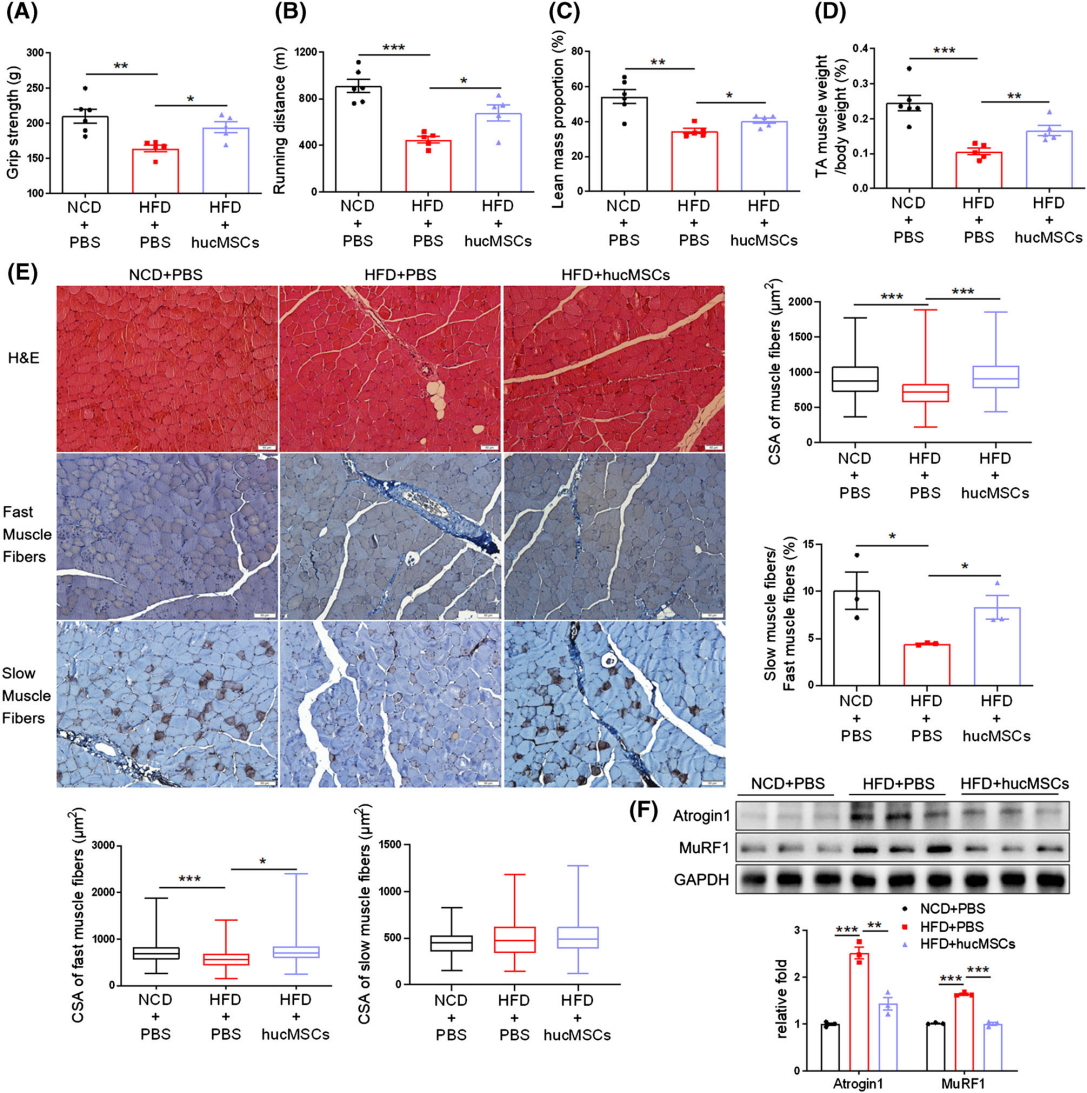
hucMSCs alleviate obesity‐induced muscle atrophy. (A) Grip strength (*n* = 5–6 mice). (B) Running distance evaluated by exhaustive running test (*n* = 5–6 mice). (C) Lean mass proportion detected by DXA (*n* = 5–6 mice). (D) Percentage of TA muscle weight in body weight (*n* = 5–6 mice). (E) H&E staining and immunohistochemical staining of fast and slow myosin heavy chain of TA muscles (scale bar, 50 μm). And cross‐sectional area (CSA) of muscle fibres, fast and slow muscle fibres and the ratio of slow muscle fibres to fast muscle fibres (*n* = 3–5 mice). (F) Western blot analysis of Atrogin 1 and MuRF1 in TA muscles (*n* = 3 mice). Quantification of bands was performed using ImageJ software. Data are mean ± SEM. (*P < 0.05, **P < 0.01, ***P < 0.001 by two‐sided unpaired Student's *t*‐test).

To further explore the role of hucMSCs in regulating obesity‐induced muscle atrophy, we also intravenously injected hucMSCs into mice fed with a high‐fat diet (HFD). HFD was started at 8 weeks old, whereas the hucMSC injection (1 × 10^6^ cells per mouse suspended in 200 μL PBS) or PBS control (200 μL per mouse) was started at 38 weeks old via the tail vein every 7 days for a total of 8 injections. One week after the last administration, hucMSCs improved glucose and insulin tolerance (*Figure* *S3* *A‐B*), grip strength (*Figure* 2A), and treadmill running endurance (*Figure* 2B). hucMSCs also reduced HFD‐induced body weight gain (*Figure* *S3* *C*) but did not affect lean mass (*Figure* *S3* *D*), whereas elevated lean mass proportion (*Figure* 2C), and increased TA muscle mass (*Figures* 2D and S3E). Meanwhile, hucMSCs also increased the CSA of muscle fibres, especially fast muscle fibres, elevated the percentage of slow in fast muscle fibres and the number of muscle fibres (*Figures* 2E and S3F), and suppressed obesity‐associated upregulation of Atrogin1 and MuRF1 protein levels (*Figure* 2F). Additionally, muscle fibrosis in HFD mice was also ameliorated by hucMSCs (*Figure* *S3* *G*). These results indicate that hucMSCs alleviate obesity‐induced muscle atrophy.

### 3.3 hucMSCs ameliorate IM‐induced muscle atrophy

**Figure 3:**
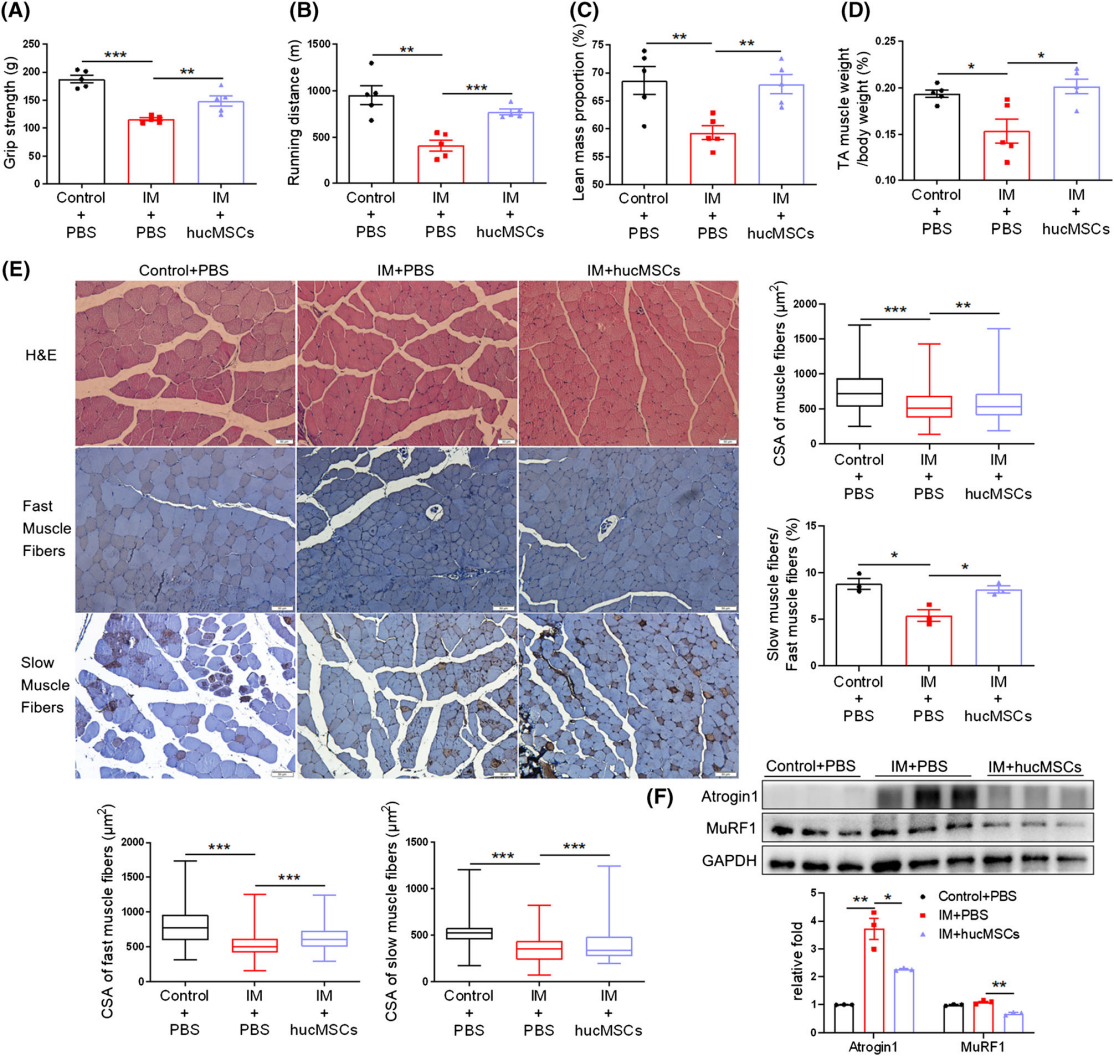
hucMSCs ameliorate immobilization (IM)‐induced muscle atrophy. (A) Grip strength (*n* = 5 mice). (B) Running distance evaluated by exhaustive running test (*n* = 5 mice). (C) Lean mass proportion detected by DXA (*n* = 5 mice). (D) Percentage of TA muscle weight in body weight (*n* = 5 mice). (E) H&E staining and immunohistochemical staining of fast and slow myosin heavy chain of TA muscles (scale bar, 50 μm). And cross‐sectional area (CSA) of muscle fibres, fast and slow muscle fibres and the ratio of slow muscle fibres to fast muscle fibres (*n* = 3–5 mice). (F) Western blot analysis of Atrogin 1 and MuRF1 in TA muscles (*n* = 3 mice). Quantification of bands was performed using ImageJ software. Data are mean ± SEM. (*P < 0.05, **P < 0.01, ***P < 0.001 by two‐sided unpaired Student's *t*‐test).

It is unknown whether the observed anti‐atrophy effects of systemic hucMSC administration are secondary to improved glucose homeostasis in diabetes and obesity mouse models. We sought to use the immobilization (IM)‐induced disuse muscle atrophy model to address this question. Mice were immobilized for 2 weeks^S1,S6^ using soft plastic‐coated wire ties, and the fixed limbs were locally injected with hucMSCs (1 × 10^6^ cells per mouse suspended in 200 μL PBS) or PBS control every 7 days for a total of 4 injections. hucMSC intervention ameliorated grip strength (*Figure* 3A), and treadmill running endurance (*Figure* 3B). hucMSCs did not affect lean mass (*Figure* S4A), but upregulated lean mass proportion (*Figure* 3C) and TA muscle mass (*Figures* 3D and S4B). hucMSCs also increased the CSA of muscle fibres, including fast and slow muscle fibres in IM mice, elevated the percentage of slow in fast muscle fibres and the number of muscle fibres (*Figures* 3E and S4C), and reduced IM‐induced upregulation of Atrogin1 and MuRF1 expression (*Figure* 3F). Additionally, muscle fibrosis in IM mice was also alleviated by hucMSCs (*Figure* *S4* *D*). The results suggest that the anti‐atrophy effects of hucMSCs are likely due to their direct effects on muscles rather than the indirect effects through systemic metabolism.

### 3.4 hucMSCs rescue atrophy‐associated impairment of the AMPK/ULK1 signalling and autophagy

**Figure 4:**
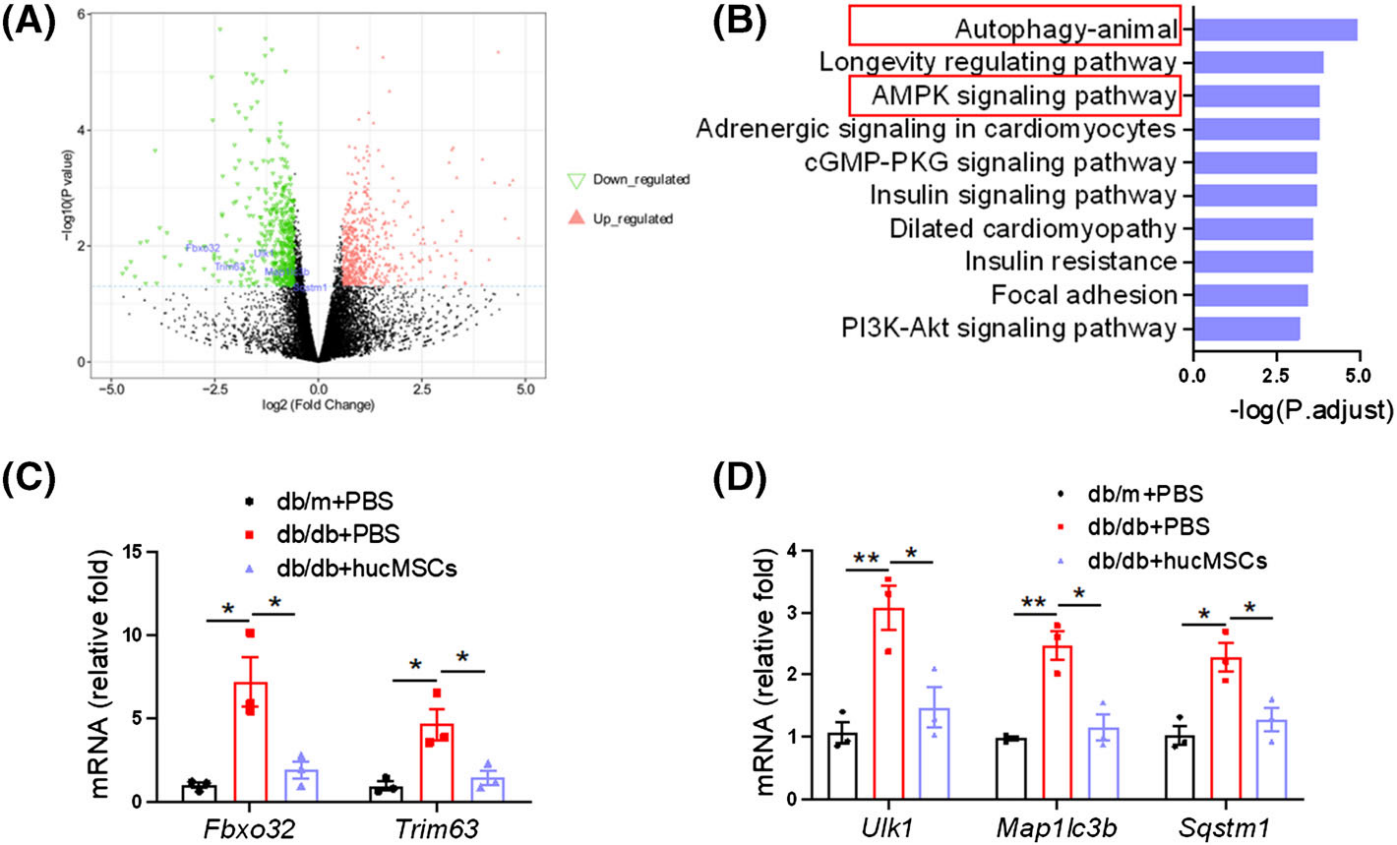
RNA‐seq analysis of TA muscle from db/db mice. (A) The volcano map of differentially expressed genes (hucMSCs vs. PBS) identified by RNA‐seq analysis of TA muscle from db/db mice with PBS or hucMSCs treatment (*n* = 3 mice). (B) KEGG analysis of the enrichment pathways. (C) RT‐qPCR validation of *Fbxo32(Atrogin1)* and *Trim63(MuRF1)* (*n* = 3 mice). (D) RT‐qPCR validation of *Ulk1*, *map 1lc3b* and *Sqstm1* (*n* = 3 mice). Data are mean ± SEM. (*P < 0.05, **P < 0.01 by two‐sided unpaired Student's *t*‐test).

**Figure 5:**
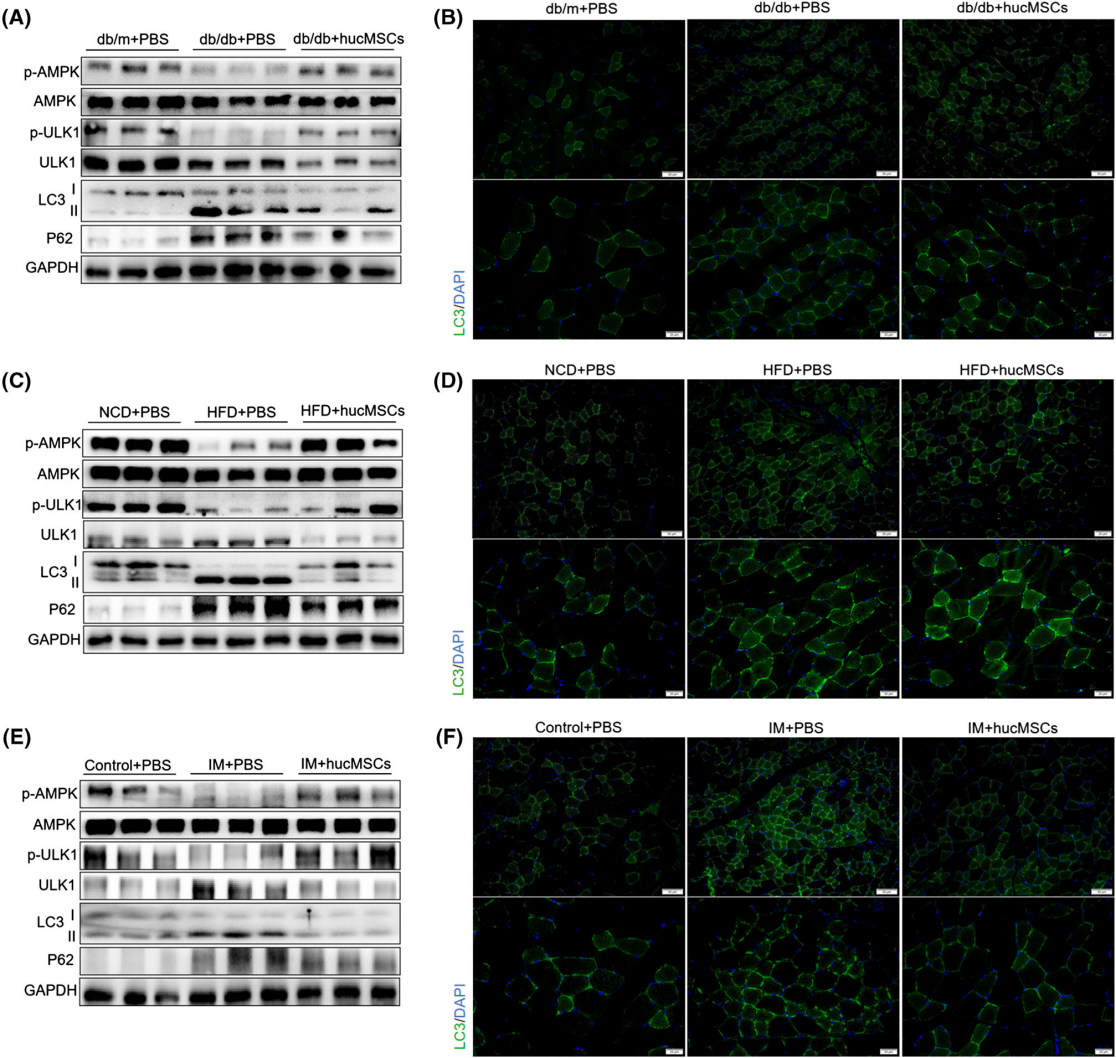
hucMSCs rescue atrophy‐associated impairment of the AMPK/ULK1 signalling and autophagy. (A) Western blot analysis of p‐AMPK (T172) and p‐ULK1 (S555), LC3 and p62 in TA muscles of db/db mice (*n* = 3 mice). (B) Immunofluorescence staining of LC3 in TA muscles of db/db mice (scale bar, 50 and 20 μm). (C) Western blot analysis of p‐AMPK (T172) and p‐ULK1 (S555), LC3 and p62 in TA muscles of HFD mice (*n* = 3 mice). (D) Immunofluorescence staining of LC3 in TA muscles of HFD mice (scale bar, 50 and 20 μm). (E) Western blot analysis of p‐AMPK (T172) and p‐ULK1 (S555), LC3 and p62 in TA muscles of IM mice (*n* = 3 mice). (F) Immunofluorescence staining of LC3 in TA muscles of IM mice (scale bar, 50 and 20 μm).

To explore the mechanism underlying hucMSC‐mediated alleviation of muscle atrophy, we performed RNA‐seq analysis of the TA muscle from db/db mice with PBS or hucMSC injections. We identified 1426 differentially expressed genes (DEGs); 627 genes were upregulated in hucMSCs vs. PBS, and 799 genes were downregulated (*Figure* 4A). KEGG pathway analysis revealed that DEGs were enriched in AMPK signalling and autophagy (*Figure* 4B). RT‐qPCR analysis validated that hucMSC injection downregulated muscle atrophy related genes *Fbxo32(Atrogin1)*, *Trim63(MuRF1)* (*Figure* 4C) and autophagy related genes *Ulk1*, *Map 1lc3b* and *Sqstm1* (*Figure* 4D). In db/db mice, the phosphorylation of AMPK(T172) and downstream ULK1(S555) were lower than in db/m mice, which was upregulated by hucMSC injection (*Figures* 5A and S5A). Meanwhile, the ratio of LC3‐II to LC3‐I and the p62 content were significantly higher in db/db mice than in db/m mice but were obviously down‐regulated by hucMSC intervention (*Figures* 5A and S5A). In addition, immunofluorescence staining of LC3 showed that hucMSC injection decreased the proportion of LC3‐positive muscle fibres, which indicated that hucMSCs elevated the level of autophagy and restored autophagy flux in skeletal muscle (*Figures* 5B and S5B). In HFD mice and IM mice, hucMSC intervention also promoted phosphorylation of AMPK (T172) and ULK1 (S555), and reduced the ratio of LC3‐II to LC3‐I, the p62 content and the proportion of LC3‐positive muscle fibres (*Figures* 5C–F and S5C–F). These results demonstrate that hucMSCs activate AMPK/ULK1 signalling and promote autophagy in skeletal muscles.

### 3.5 hucMSCs alleviate PA‐induced C2C12 myotube atrophy via promoting AMPK‐mediated autophagy

**Figure 6:**
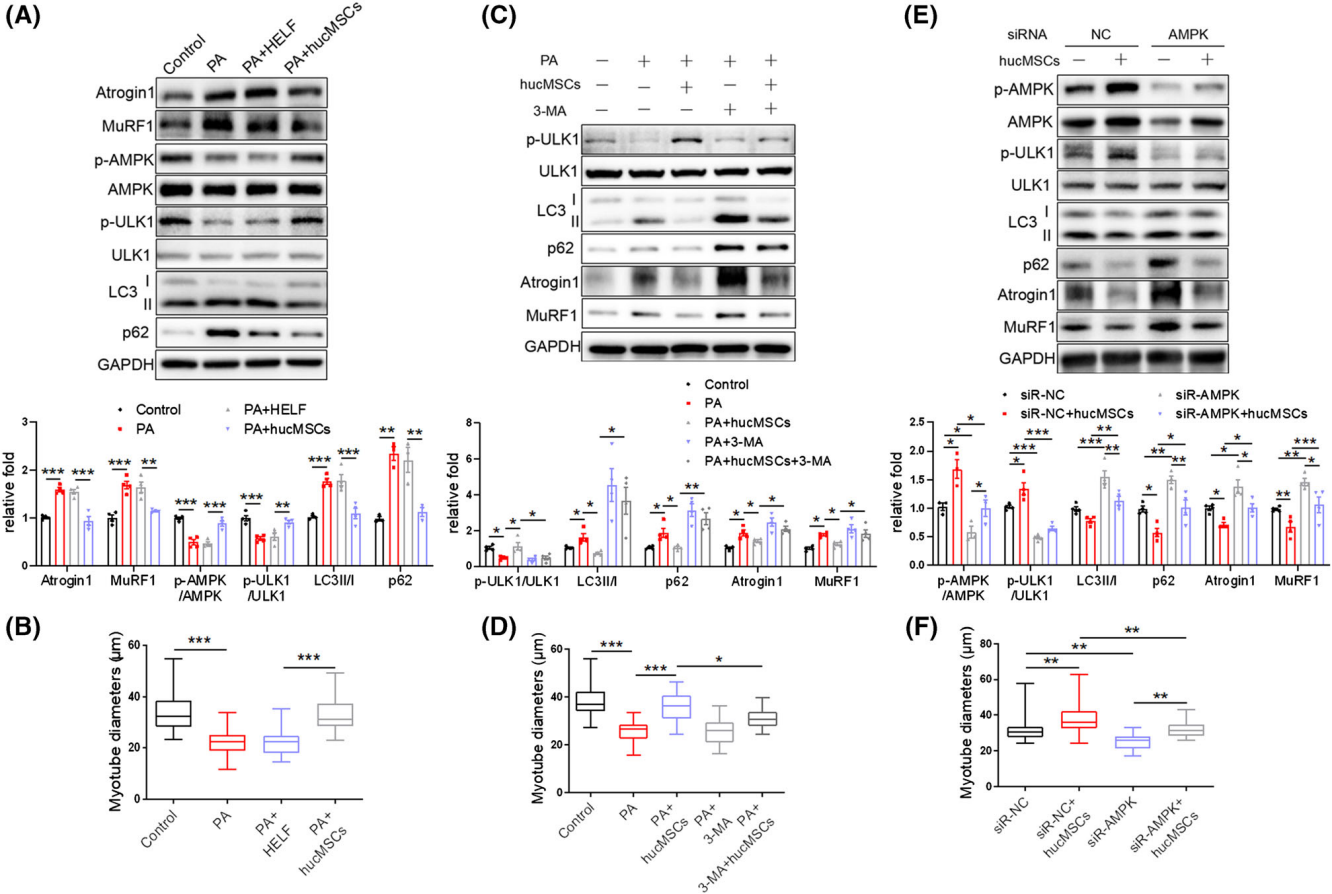
hucMSCs alleviate PA‐induced C2C12 myotube atrophy via promoting AMPK‐mediated autophagy. (A) Western blot analysis of Atrogin 1 and MuRF1, p‐AMPK (T172) and p‐ULK1 (S555), LC3 and p62 in C2C12 myotubes treated with PA and hucMSCs (*n* = 3–4). (B) Diameters of C2C12 myotubes treated with PA and hucMSCs. (C) Western blot analysis of p‐ULK1(S555), LC3 and p62, Atrogin 1 and MuRF1 in C2C12 myotubes treated with hucMSCs and autophagy inhibitor 3‐MA (*n* = 4). (D) Diameters of C2C12 myotubes treated with hucMSCs and 3‐MA. (E) Western blot analysis of p‐AMPK (T172) and p‐ULK1 (S555), LC3 and p62, Atrogin1 and MuRF1 in C2C12 myotubes transfected with AMPK siRNA and treated with hucMSCs (*n* = 3–4). (F) Diameters of C2C12 myotubes transfected with AMPK siRNA and treated with hucMSCs. Quantification of bands was performed using ImageJ software. Data are mean ± SEM. (*P < 0.05, **P < 0.01, ***P < 0.001 using one‐way ANOVA followed by Tukey's test).

To further characterize the direct cell‐autonomous effect of hucMSCs on muscles, we turned to the *in vitro* C2C12 myocyte model. C2C12 myoblasts were fully differentiated and treated with 0.6 mM palmitate (PA) to induce atrophic signalling. PA is known to mimic lipotoxicity and induced atrophy.^S7,S8^ At this dosage, PA treatment robustly promoted the expression of Atrogin 1 and MuRF1, whereas hucMSC treatment abrogated PA‐induced increase of Atrogin 1 and MuRF1 expression (*Figure* 6A). Meanwhile, PA reduced phosphorylation of AMPK (T172) and ULK1 (S555), and elevated the ratio of LC3‐II to LC3‐I and the p62 content in the myotubes. hucMSC treatment significantly rescued AMPK/ULK1 signalling transduction and decreased LC3II/LC3I and p62 content, suggesting enhanced autophagy process and autophagy flux (*Figure* 6A). hucMSC treatment also increased diameters of myotubes (*Figures* 6B and S6A). These results support the direct cell‐autonomous effects of hucMSCs on myocytes. To further address whether AMPK‐mediated autophagy is required for the effects of hucMSCs on myocytes, we used autophagy inhibitor 3‐MA or siRNA targeting AMPK. hucMSCs‐mediated upregulation of ULK1 phosphorylation and reduction of LC3II/LC3I and p62 content were partially abolished in the presence of 3‐MA (*Figure* 6C). 3‐MA also diminished hucMSCs‐dependent decrease of Atrogin 1 and MuRF1 levels (*Figure* 6C) and increase of myotube diameters (*Figures* 6D and S6B). AMPK knockdown with siRNA mimicked 3‐MA in diminishing hucMSC effects in p‐AMPK/p‐ULK1, LC3/p62, Atrogin1/MuRF1 and myotube diameters (*Figures* 6E,F and S6C). These results suggest that hucMSCs alleviate PA‐induced muscle atrophy by promoting AMPK‐mediated autophagy in C2C12 myotubes.

### 3.6 MSC‐EXO alleviates diabetes‐associated muscle atrophy and enhances the AMPK/autophagy signalling

**Figure 7:**
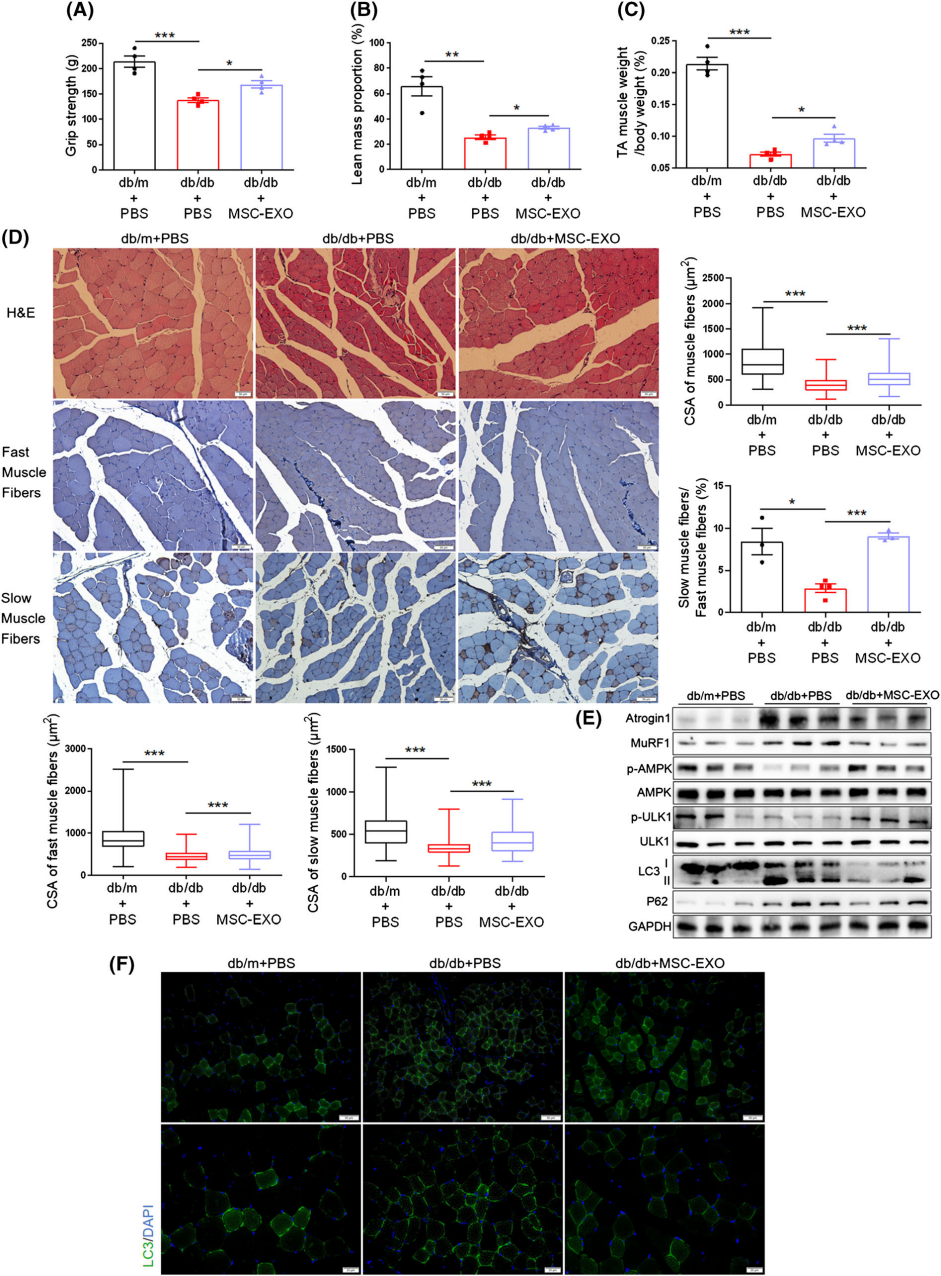
MSC‐EXO alleviates diabetes‐associated muscle atrophy and enhances the AMPK/autophagy signalling. (A) Grip strength (*n* = 4 mice). (B) Lean mass proportion detected by DXA (*n* = 4 mice). (C) Percentage of TA muscle weight in body weight (*n* = 4 mice). (D) H&E staining and immunohistochemical staining of fast and slow myosin heavy chain of TA muscles (scale bar, 50 μm). And cross‐sectional area (CSA) of muscle fibres, fast and slow muscle fibres and the ratio of slow muscle fibres to fast muscle fibres (*n* = 3–4 mice). (E) Western blot analysis of Atrogin 1 and MuRF1, p‐AMPK (T172) and ULK1(S555), LC3 and p62 in TA muscles (*n* = 3 mice). (F) Immunofluorescence staining of LC3 in TA muscles of db/db mice (scale bar, 50 and 20 μm). Data are mean ± SEM. (*P < 0.05, **P < 0.01, ***P < 0.001 by two‐sided unpaired Student's *t*‐test).

Accumulating evidence suggests that MSC therapy is due to its paracrine action rather than its differentiation mechanism.^28^, ^29^ As an important paracrine factor, exosomes contain different kinds of MSC‐derived bioactive molecules and have been considered to represent a promising therapy.^30^ ^,S4^ Therefore, we tested the effects of hucMSC‐derived exosomes (MSC‐EXO) on muscle atrophy in db/db mice. MSC‐EXO isolated from hucMSC‐conditioned medium exhibited cup‐shaped vesicles with a diameter of approximately 130 nm, as characterized by nanoparticle tracking analysis (*Figure* *S7* *A*) and transmission electron microscopy (TEM) (*Figure* *S7* *B*). Western blot showed that the protein markers, CD9, CD63, and CD81, were enriched in MSC‐EXO, whereas calnexin, an endoplasmic reticulum marker, was enriched in hucMSCs but absent in MSC‐EXO (*Figure* *S7* *C*). MSC‐EXO (200 μg dissolved in 200 μL PBS) was injected into db/db mice through the tail vein every 3 days for 8 weeks;^S9,S10^ 200 μL per mouse PBS injection served as the control. MSC‐EXO at 200 μg/mouse was previously used to improve the targeting of antitumor drugs and the intervention of diabetic foot wounds, and acquired good results.^S9,S11^ To detect the distribution of MSC‐EXO *in vivo*, MSC‐EXO was labelled with Cy7 and injected into mice. After 12 h, IVIS images showed that MSC‐EXO could reach skeletal muscle (*Figure* *S7* *D‐E*). As we expected, MSC‐EXO injection ameliorated glucose and insulin tolerance (*Figure* *S8* *A‐B*) and grip strength (*Figure* 7A). MSC‐EXO intervention did not affect body weight (*Figure* *S8* *C*), but elevated lean mass and lean mass proportion (*Figures* 7B and S8D) and increased TA muscle mass (*Figures* 7C and S8E). Additionally, MSC‐EXO also increased the CSA of muscle fibres, including fast and slow muscle fibres in db/db mice, elevated the percentage of slow in fast muscle fibres and the number of muscle fibres (*Figures* 7D and S8F). Meanwhile, MSC‐EXO suppressed diabetes‐associated upregulation of Atrogin1 and MuRF1 protein levels, promoted phosphorylation of AMPK(T172) and ULK1(S555), and reduced LC3II/LC3I and p62 content (*Figures* 7E and S8G) and the proportion of LC3‐positive muscle fibres (*Figures* 7F and S8H). These results suggest that MSC‐EXO injection protects against diabetes‐induced muscle atrophy, which is consistent with the roles of hucMSCs.

### 3.7 MSC‐EXO counteracts PA‐induced myotube atrophy by enhancing the AMPK/autophagy signalling

**Figure 8:**
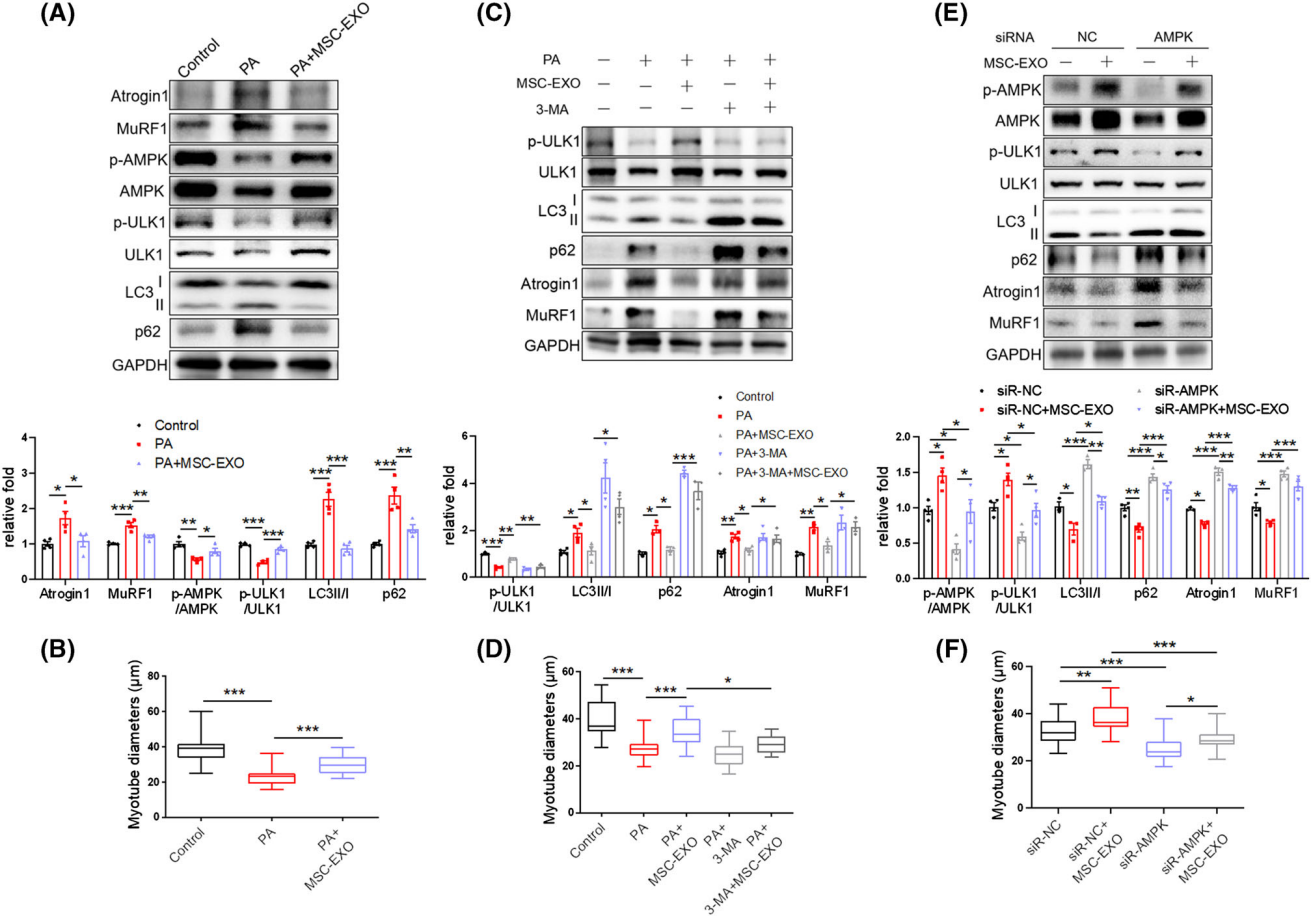
MSC‐EXO counteracts PA‐induced myotube atrophy by enhancing the AMPK/autophagy signalling. (A) Western blot analysis of Atrogin 1 and MuRF1, p‐AMPK (T172) and p‐ULK1 (S555), LC3 and p62 in C2C12 myotubes treated with PA and MSC‐EXO (*n* = 4). (B) Diameters *of* C2C12 myotubes treated with PA and MSC‐EXO. (C) Western blot analysis of p‐ULK1(S555), LC3, and p62, Atrogin 1 and MuRF1 in C2C12 myotubes treated with MSC‐EXO and autophagy inhibitor 3‐MA (*n* = 3–4). (D) Diameters of C2C12 myotubes treated with MSC‐EXO and 3‐MA. (E) Western blot analysis of p‐AMPK (T172) and p‐ULK1 (S555), LC3 and p62, Atrogin1 and MuRF1 in C2C12 myotubes transfected with AMPK siRNA and treated with MSC‐EXO (*n* = 3–4). (F) Diameters of C2C12 myotubes transfected with AMPK siRNA and treated with MSC‐EXO. Quantification of bands was performed using ImageJ software. Data are mean ± SEM. (*P < 0.05, **P < 0.01, ***P < 0.001 using one‐way ANOVA followed by Tukey's test).

To further address the cell‐autonomous mechanism of exosomes, we treated fully‐differentiated C2C12 myotubes with 25 μg/mL MSC‐EXO or PBS as the control for 24 h in the presence or absence of 3‐MA or siRNA targeting AMPK. The dosage of MSC‐EXO was based on previous literature, which demonstrated that this dosage did not affect cell viability.^S12^ We firstly labelled MSC‐EXO with PKH67 and found that labelled exosomes could be taken up by C2C12 myotubes using a fluorescence microscope (*Figure* *S9* *A*). MSC‐EXO treatment decreased PA‐induced Atrogin 1 and MuRF1 expression, rescued phosphorylation of AMPK(T172) and ULK1(S555), and reduced LC3II/LC3I and p62 content (*Figure* 8A). MSC‐EXO treatment also increased myotube diameters (*Figures* 8B and S9B). The MSC‐EXO‐mediated upregulation of ULK1 phosphorylation and reduction of LC3II/LC3I and p62 content were partially abolished by 3‐MA (*Figure* 8C). 3‐MA also diminished the MSC‐EXO‐dependent decrease of Atrogin 1 and MuRF1 levels (*Figure* 8C) and increase of myotube diameters (*Figures* 8D and S9C). AMPK knockdown with siRNA mimicked 3‐MA in diminishing MSC‐EXO effects in p‐AMPK/p‐ULK1, LC3/p62, Atrogin1/MuRF1 and myotube diameters (*Figures* 8E,F and S9D). These results indicate that MSC‐EXO mitigates muscle atrophy via promoting AMPK‐mediated autophagy.

## 4 Discussion

As a promising intervention, MSC‐based therapy has been reported to play an important role in muscle atrophy resulting from multiple diseases. For example, BM‐MSCs prevented HFD‐induced skeletal muscle wasting^25^ and immobilization‐induced muscle atrophy.^S1^ AD‐MSCs and ESCs delayed muscle atrophy after nerve injury.^S2,S3^ However, the effect of hucMSCs on muscle atrophy in T2DM is unclear. Therefore, we injected hucMSCs into db/db mice to investigate their effect on diabetes‐associated muscle atrophy. hucMSC intervention significantly increased muscle strength and prevented loss of muscle mass in db/db mice. This is the first demonstration of a role for hucMSCs in diabetes‐related muscle atrophy.

While ameliorating muscle atrophy, hucMSCs also improved glucose tolerance and insulin tolerance of mice. It is reported that insulin resistance in T2DM is a key contributor to muscle atrophy,^9^ which made us wonder whether the effect of hucMSCs on muscle atrophy is a direct effect or an indirect effect through ameliorating insulin resistance. Previous studies suggested some direct effects of MSCs on muscle function in a critical limb ischemia model and an amyotrophic lateral sclerosis model via intramuscular injection of MSCs.^33^, ^34^ In this study, we established a muscle disuse atrophy model through IM and injected hucMSCs intramuscularly. Our results showed that hucMSC intervention elevated muscle mass and enhanced muscle strength in IM mice, suggesting that hucMSCs likely have direct effects on diabetes‐associated muscle atrophy.

What is the mechanism for MSC‐mediated anti‐atrophy effects? Given their ability to differentiate into myocytes, MSCs were thought to be directly incorporated into muscle tissues. However, previous studies did not observe MSCs incorporation into the muscle tissue of HFD mice and IM mice.^25^, ^27^ Although MSCs have the ability to differentiate into multiple cell types,^35^ there is controversy over the localization, survival, and persistence of MSCs *in vivo* after administration. The route of administration is an important factor in determining the fate of MSCs. Intravenous injection has become the most commonly used route of administration, which is safe and allows the administration of large amounts of MSCs. However, *in vivo* tracking study found that the majority of MSCs localized to the lungs after intravenous injection, whereas a few entered the liver, spleen, or damaged tissues, and the survival time did not exceed 24 h.^36^ However, the therapeutic benefits associated with MSCs administration often persist longer than the survival time of MSCs themselves. It was originally believed that administered MSCs could migrate to the sites of injury, engraft and differentiate into functional cells, leading to the regeneration of damaged or diseased connective tissues. But it became increasingly evident that transplanted MSCs were not recruited to damaged tissues in large numbers or survived long enough *in vivo*.^28^, ^29^ In contrast, accumulating studies suggest that MSCs have the capacity to maintain the growth and viability of certain cell types by secreting trophic factors.^28^, ^29^ Our previous studies also demonstrated that MSCs ameliorate pancreatic α‐cell and endothelium function in diabetes through paracrine factors.^31^, ^37^ Exosomes secreted by MSCs contain multiple bioactive molecules, which have been considered to represent a promising therapy.^30^ ^,S4^ In the current study, we found that Cy7‐labelled MSC‐EXO could successfully reach skeletal muscle of mice via IVIS images. MSC‐EXO intervention increased muscle strength and muscle mass of db/db mice, which coincides with the effects of hucMSCs. Meanwhile, MSC‐EXO could easily be taken up by C2C12 myotubes *in vitro* and ameliorated PA‐induced muscle atrophy. Our results demonstrated that hucMSCs ameliorate diabetes‐associated muscle atrophy through exosomes, although molecules in exosomes underlying these effects remain to be identified.

Autophagy plays is critical for maintaining homeostasis of muscle mass under physiological and pathological conditions.^15^ Autophagy deficiency affects organelle shaping mechanisms and leads to the accumulation of abnormal mitochondria and dilated sarcoplasmic reticulum. In skeletal muscle, the accumulation of dysfunctional organelles activates intracellular catabolism, leading to muscle atrophy and weakness.^15^ The function of autophagy declines during aging, and promoting basal autophagy ameliorates age‐related muscle dysfunction by increasing the selective degradation of misfolded proteins or dysfunctional organelles.^38^ Conversely, autophagy inhibition leads to aging‐related muscle atrophy, characterized by accumulation of LC3 and p62.^16^ Muscle‐specific deletion of the Atg7 gene in adulthood leads to autophagy deficiency with accumulation of p62, causing muscle loss and weakness.^15^ Previous studies have shown that autophagy flux levels are altered in diabetic skeletal muscle.^14^ The autophagy markers LC3 and p62 were accumulated in the muscles of db/db mice, which confirmed that autophagic flux is inhibited under diabetic conditions.^39^ In the current study, we also obtained similar results. The enhanced autophagy process and autophagy flux in muscles treated with hucMSCs or MSC‐EXO in our study supports that autophagy improves muscle atrophy, which is consistent with the role of MSCs in enhancing autophagy in other target organs.^S13,S14^

AMPK is an important intracellular energy sensor regulating metabolic homeostasis,^21^ which is crucial for maintaining skeletal muscle function. During fasting, muscle AMPK induces autophagy and muscle protein breakdown to prevent hypoglycaemia. During aging, AMPK delays the onset of muscle myopathy and mitochondrial dysfunction. AMPK muscle‐specific deletion in aged mice impairs muscle autophagy.^18^ ULK1 serves as an important initiator in autophagy progress.^S15,S16^ AMPK is a positive regulator of autophagy through phosphorylation of ULK1 at several primary sites, including Ser555, Ser317, and Ser777.^S17^ Deletion of AMPK from skeletal muscle dramatically reduces the exercise capacity of mice,^19^ whereas AMPK activation ameliorates muscle dystrophy.^20^ Here we found that AMPK is involved in the amelioration of autophagy by hucMSCs and MSC‐EXO, which provides a new scientific basis and intervention target for MSCs or exosome to treat muscle atrophy diseases. In conclusion, our results suggest that by secreting exosomes, hucMSCs activate AMPK/ULK1‐mediated autophagy, which ameliorates muscle atrophy in diabetes and obesity. These findings demonstrate the therapeutic potential of hucMSCs and exosomes in treating muscle atrophy.

## Conflict of interest

All authors declare that they have no competing interests.

## Supporting information


**Figure S1.** Identification of hucMSCs. *(A)* Flow cytometry analysis of hucMSCs markers CD105, CD73, CD34 and HLA‐DR. *(B)* Oil Red O staining for adipogenic differentiation ability of hucMSCs (Scale bar, 20 μm). *(C)* Alizarin Red S staining for osteogenic differentiation ability of hucMSCs (Scale bar, 20 μm).
**Figure S2.** hucMSCs alleviate diabetes‐induced muscle atrophy. *(A)* Intraperitoneal glucose tolerance test (IPGTT) and area under the curve (AUC) of db/db mice after hucMSC injection (*n* = 5–6 mice). *(B)* Intraperitoneal insulin tolerance test (IPITT) and AUC of db/db mice after hucMSC injection (*n* = 5–6 mice). *(C)* Body weight (n = 5–6 mice). *(D)* Lean mass detected by Dual‐energy X‐ray absorptiometry (DXA) (n = 5–6 mice). *(E)* Tibialis anterior (TA) muscle weight (*n* = 5–6 mice). *(F)* Number of muscle fibres (*n* = 3 mice). *(G)* Masson staining and Sirius Red staining of TA muscles (Scale bar, 50 μm). Data are mean ± SEM. (*P < 0.05, **P < 0.01, ***P < 0.001 by 2‐sided unpaired student's t‐test).
**Figure S3.** hucMSCs alleviate obesity‐induced muscle atrophy. *(A)* IPGTT and AUC of HFD mice after hucMSC injection (*n* = 5–6 mice). *(B)* IPITT and AUC of HFD mice after hucMSC injection (n = 5–6 mice). *(C)* Body weight (n = 5–6 mice). *(D)* Lean mass detected by DXA (n = 5–6 mice). *(E)* TA muscle weight (*n* = 5–6 mice). *(F)* Number of muscle fibres (*n* = 3 mice). *(G)* Masson staining and Sirius Red staining of TA muscles (Scale bar, 50 μm). Data are mean ± SEM. (*P < 0.05, **P < 0.01, ***P < 0.001 by 2‐sided unpaired student's t‐test).
**Figure S4.** hucMSCs ameliorate immobilization (IM)‐induced muscle atrophy. *(A)* Lean mass detected by DXA (*n* = 5 mice). *(B)* TA muscle weight (n = 5 mice). *(C)* Number of muscle fibres (*n* = 3 mice). *(D)* Masson staining and Sirius Red staining of TA muscles (Scale bar, 50 μm). Data are mean ± SEM. (*P < 0.05, **P < 0.01, ***P < 0.001 by 2‐sided unpaired student's t‐test).
**Figure S5.** hucMSCs rescue atrophy‐associated impairment of the AMPK/ULK1 signalling and autophagy. *(A)* Relative protein‐expression levels of p‐AMPK (T172) and p‐ULK1 (S555), LC3 and p62 in TA muscles of db/db mice (*n* = 3 mice). *(B)* Proportion of LC3‐positive muscle fibres in TA muscles of db/db mice (*n* = 4 mice). *(C)* Relative protein‐expression levels of p‐AMPK (T172) and p‐ULK1 (S555), LC3 and p62 in TA muscles of HFD mice (*n* = 3 mice). *(D)* Proportion of LC3‐positive muscle fibres in TA muscles of HFD mice (*n* = 4 mice). *(E)* Relative protein‐expression levels of p‐AMPK (T172) and p‐ULK1 (S555), LC3 and p62 in TA muscles of IM mice (n = 3 mice). *(F)* Proportion of LC3‐positive muscle fibres in TA muscles of IM mice (n = 4 mice). Data are mean ± SEM. (*P < 0.05, **P < 0.01, ***P < 0.001 by 2‐sided unpaired student's t‐test).
**Figure S6.** Imaging of C2C12 myotubes. *(A)* Imaging of C2C12 myotubes treated with PA and hucMSCs (Scale bar, 50 μm). *(B)* Imaging of C2C12 myotubes treated with hucMSCs and 3‐MA (Scale bar, 50 μm). *(C)* Imaging of C2C12 myotubes transfected with AMPK siRNA and treated with hucMSCs (Scale bar, 50 μm).
**Figure S7.** Identification of MSC‐EXO and *in vivo* tracing. *(A)* Nanoparticle tracking analysis of exosomal sizes. *(B)* Transmission electron microscopy (TEM) images of MSC‐EXO. *(C)* Western blot analysis of the exosomal markers CD9, CD63, CD81, and endoplasmic reticulum marker Calnexin of MSC‐EXO. *(D)* Distribution of Cy7‐labelled MSC‐EXO in mice after 12 h of infusion by an in vivo imaging system (IVIS). *(E)* Representative Cy7 signals in dissected organs.
**Figure S8.** MSC‐EXO alleviates diabetes‐associated muscle atrophy and enhances the AMPK/autophagy signalling. *(A)* IPGTT and AUC of db/db mice after MSC‐EXO injection (*n* = 4 mice). *(B)* IPITT and AUC of db/db mice after MSC‐EXO injection (*n* = 4 mice). *(C)* Body weight (n = 4 mice). *(D)* Lean mass detected by DXA (n = 4 mice). *(E)* TA muscle weight (*n* = 4 mice). *(F)* Number of muscle fibres (*n* = 3–4 mice). *(G)* Relative protein‐expression levels of Atrogin 1 and MuRF1, p‐AMPK (T172) and p‐ULK1 (S555), LC3 and p62 in TA muscles of db/db mice (n = 3 mice). *(H)* Proportion of LC3‐positive muscle fibres in TA muscles of db/db mice (n = 4 mice). Data are mean ± SEM. (*P < 0.05, **P < 0.01, ***P < 0.001 by 2‐sided unpaired student's t‐test).
**Figure S9.** MSC‐EXO tracing in vitro and imaging of C2C12 myotubes. *(A)* Fluorescence‐tracing of PKH67‐labelled MSC‐EXO uptaken by C2C12 myotubes (Scale bar, 20 μm). *(B)* Imaging of C2C12 myotubes treated with PA and MSC‐EXO (Scale bar, 50 μm). *(C)* Imaging of C2C12 myotubes treated with MSC‐EXO and 3‐MA (Scale bar, 50 μm). *(D)* Imaging of C2C12 myotubes transfected with AMPK siRNA and treated with MSC‐EXO (Scale bar, 50 μm).

**Data S1.** Supporting Information
