# Supplementary material for: Mesenchymal stromal cells ameliorate diabetes‐induced muscle atrophy through exosomes by enhancing AMPK/ULK1‐mediated autophagy
Source: J Cachexia Sarcopenia Muscle. 2023 Jan 27;14(2):915–29. doi: 10.1002/jcsm.13177 (PMC10067482; doi:10.1002/jcsm.13177)

Supplementary Figure 1

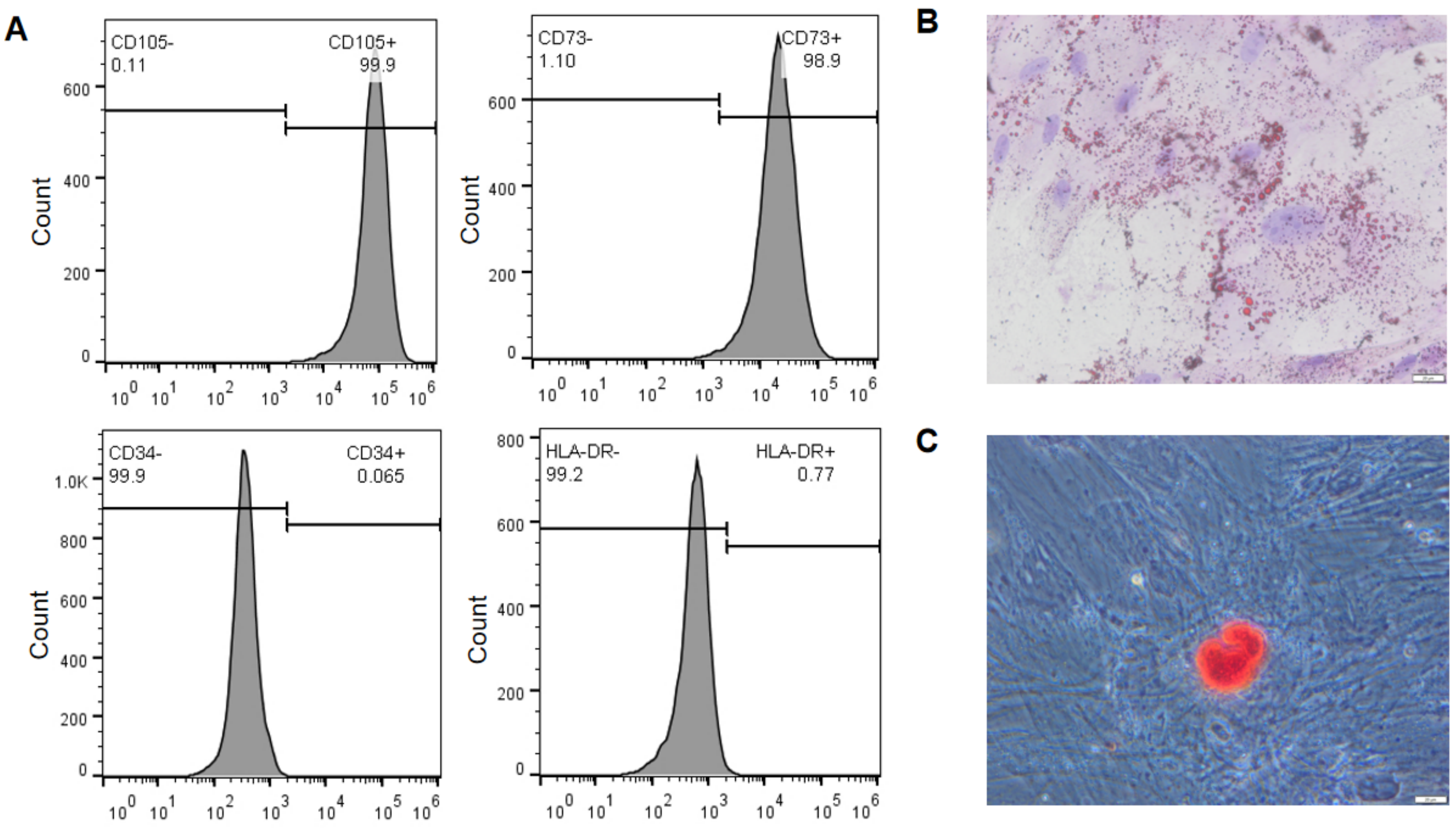

Supplementary Figure 2

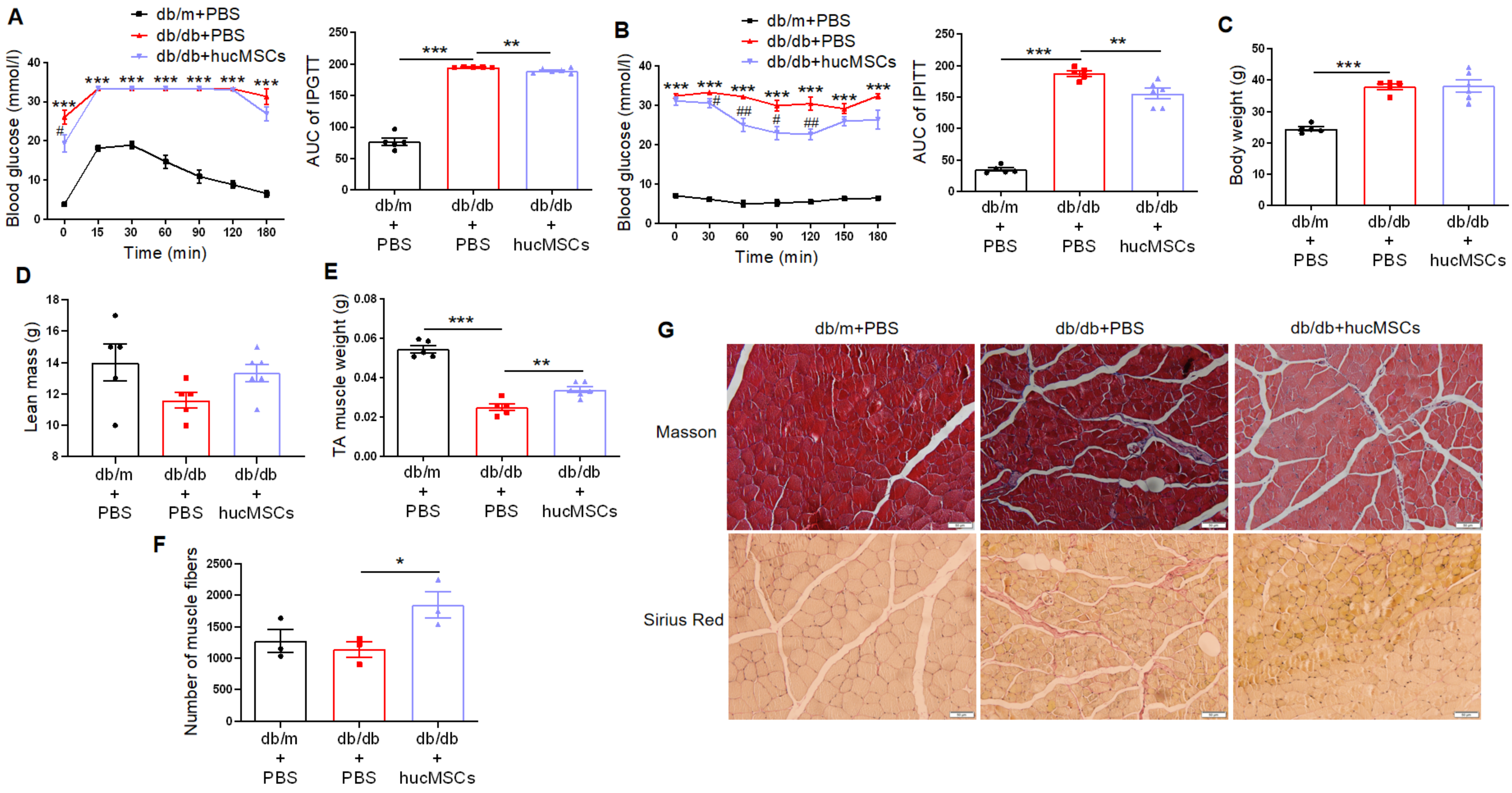

Supplementary Figure 3

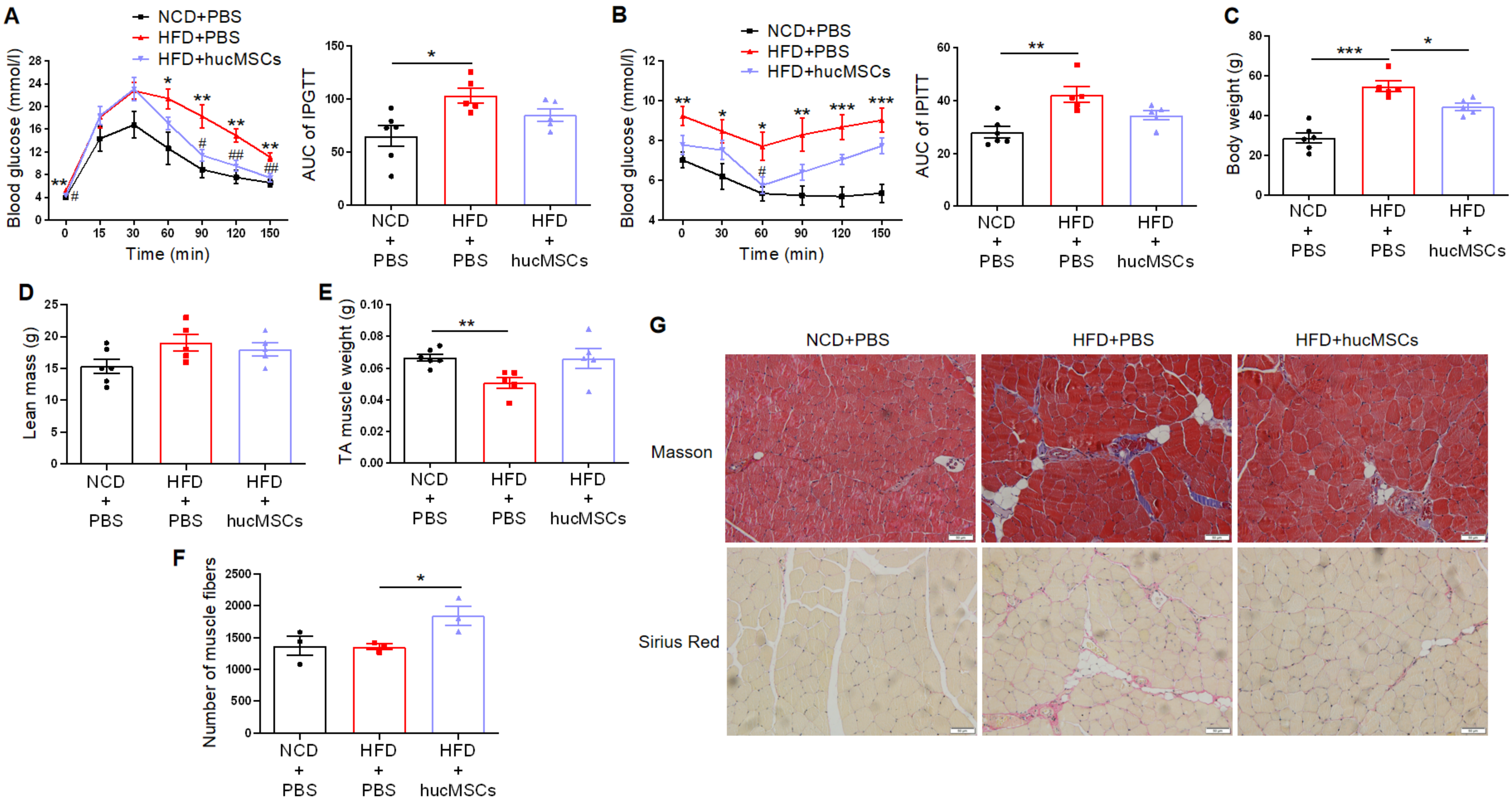

Supplementary Figure 4

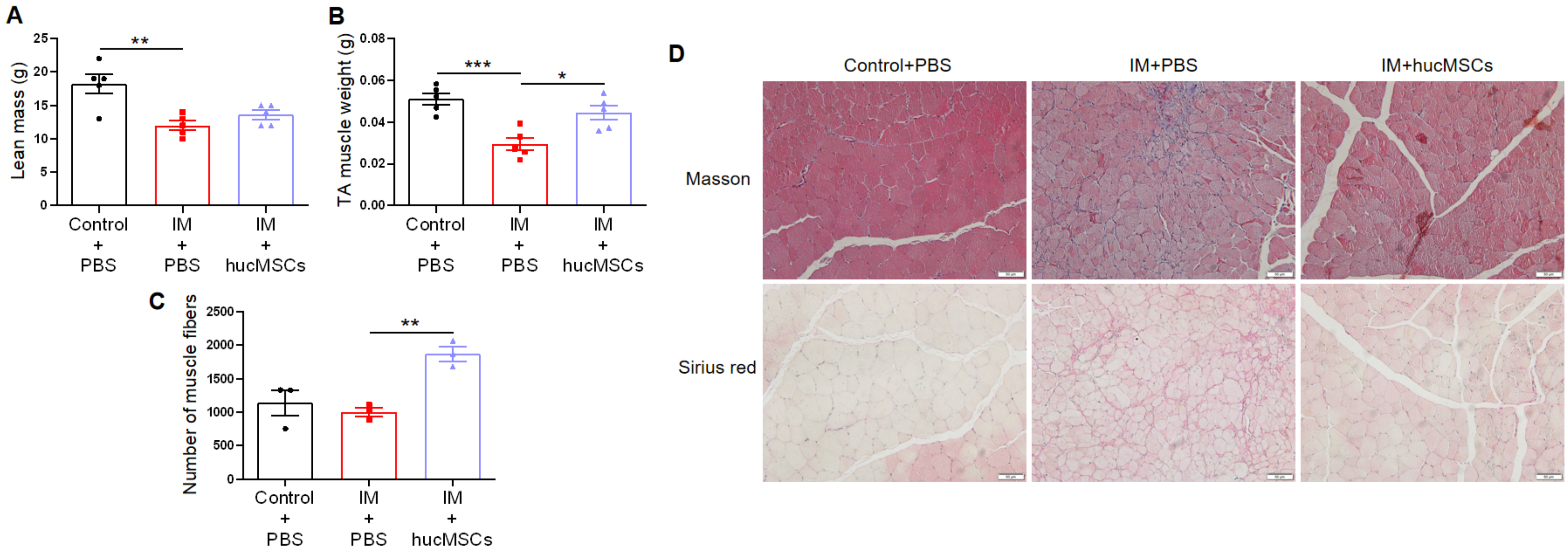

Supplementary Figure 5

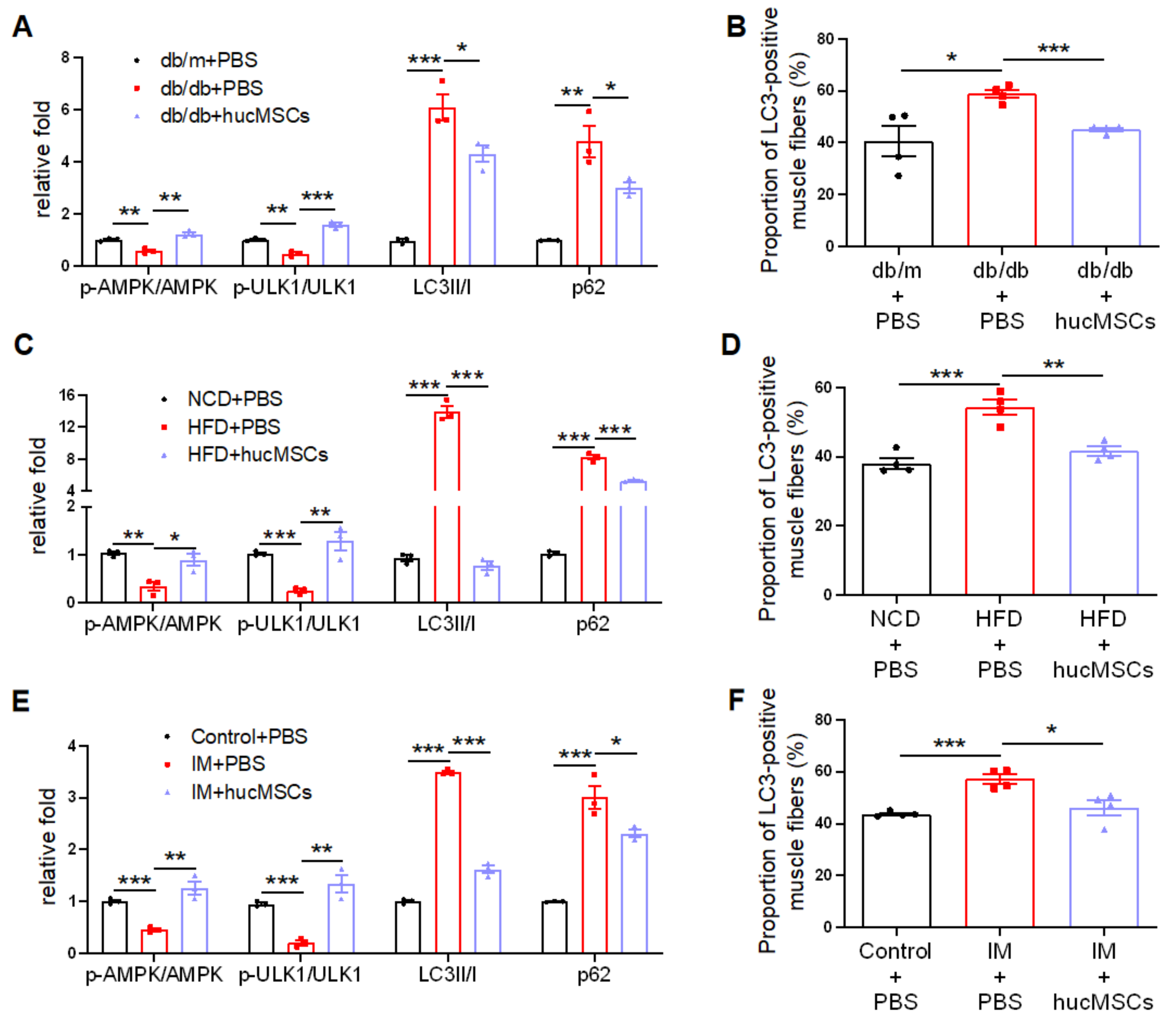

Supplementary Figure 6

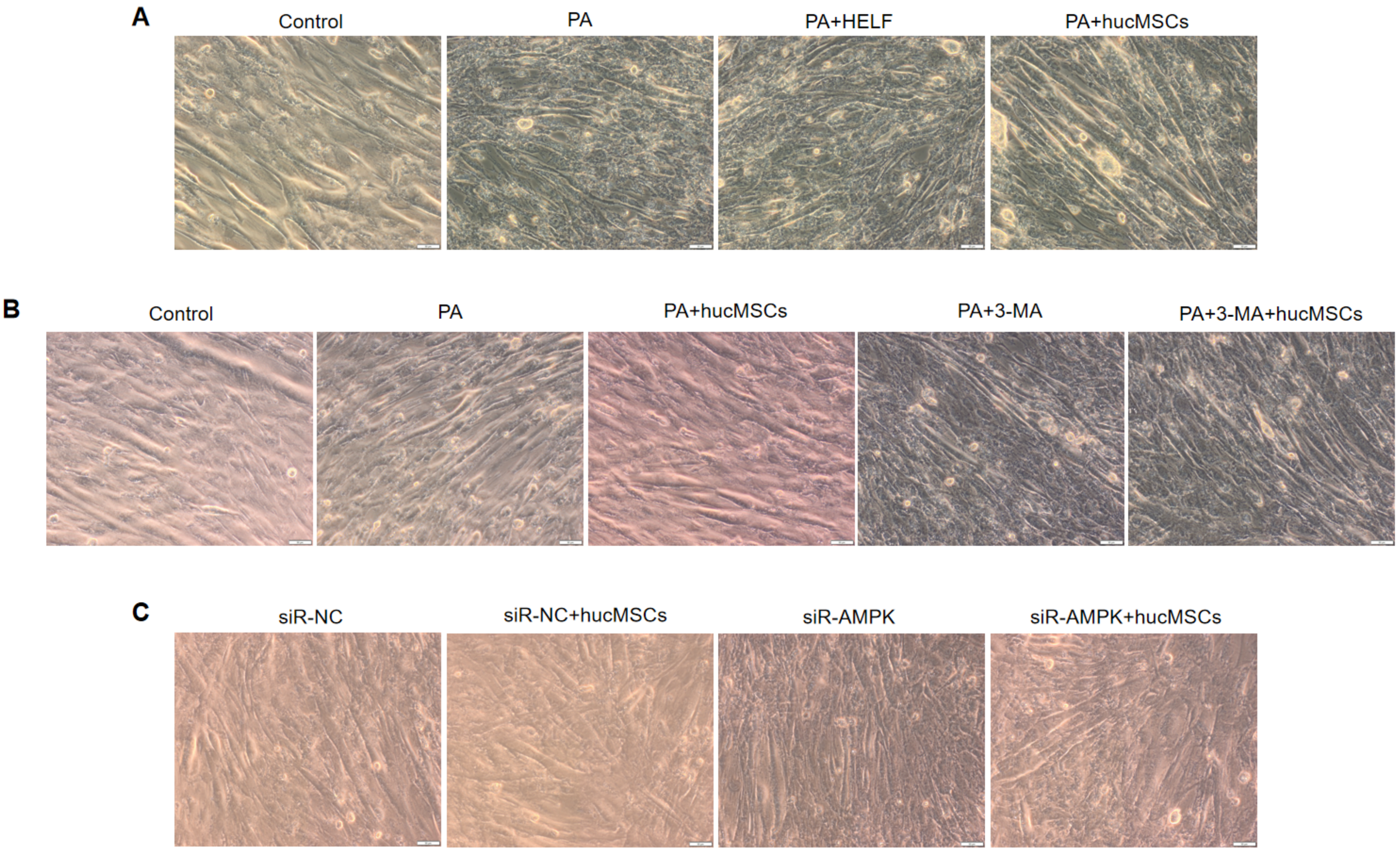

Supplementary Figure 7

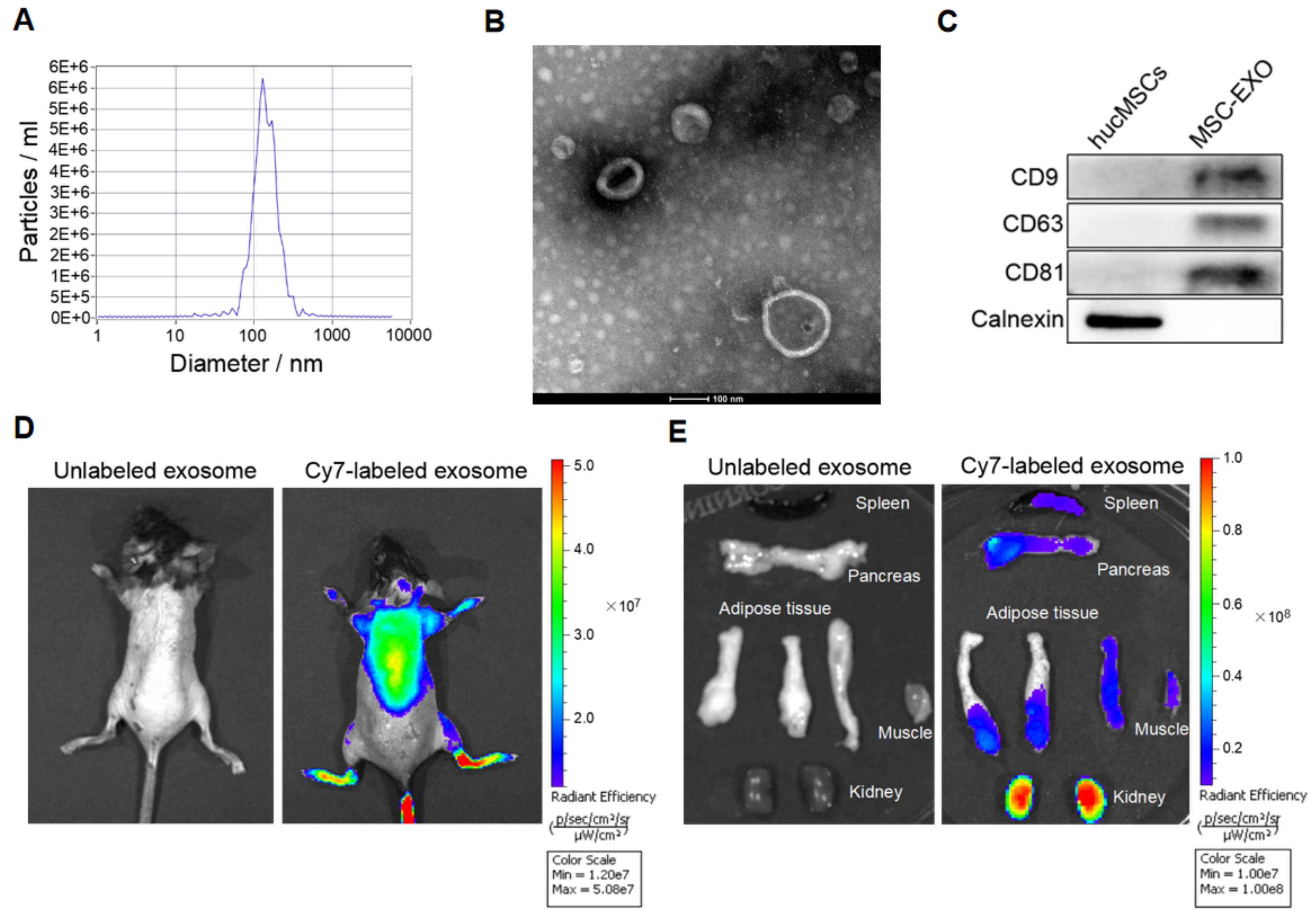

Supplementary Figure 8

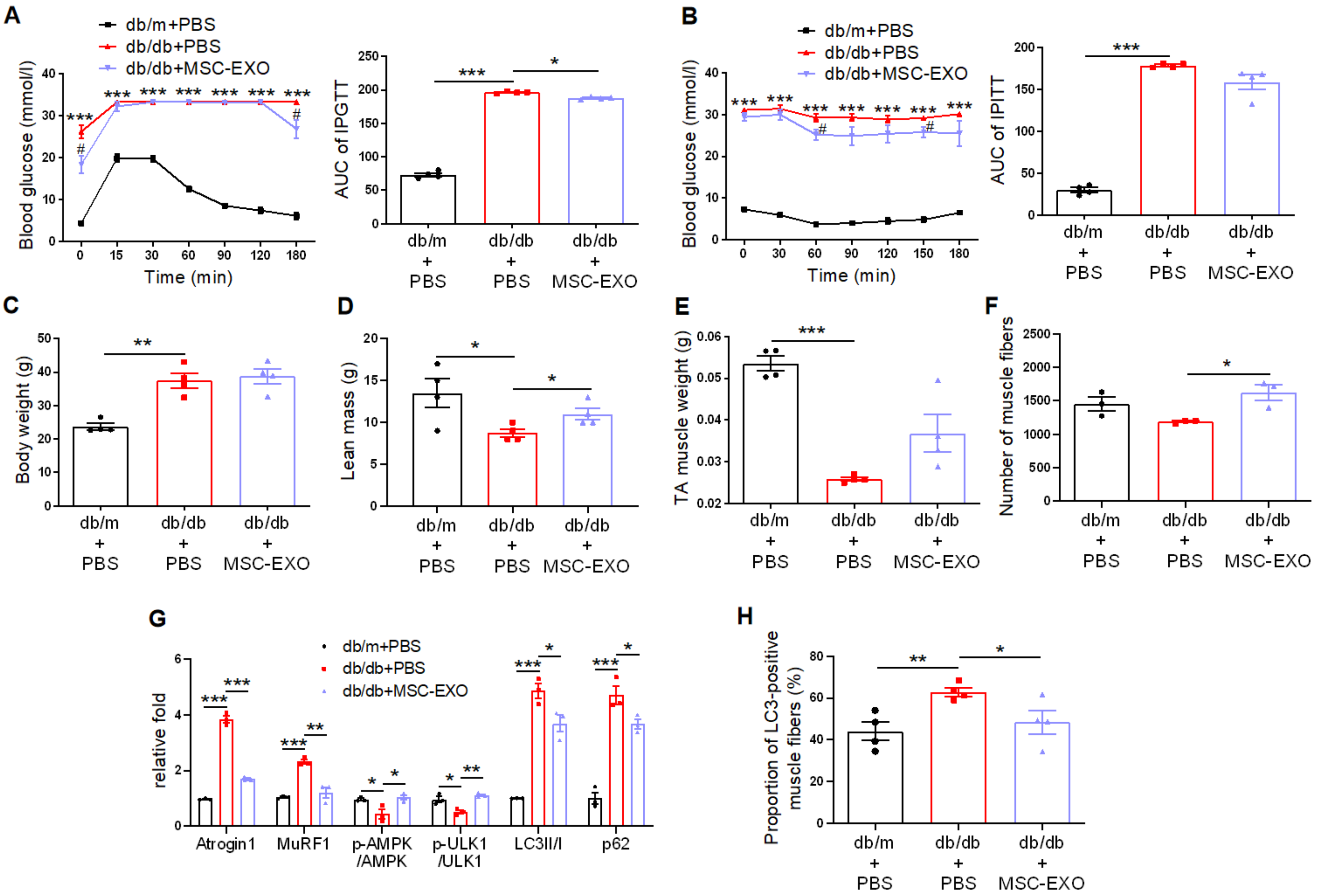

Supplementary Figure 9

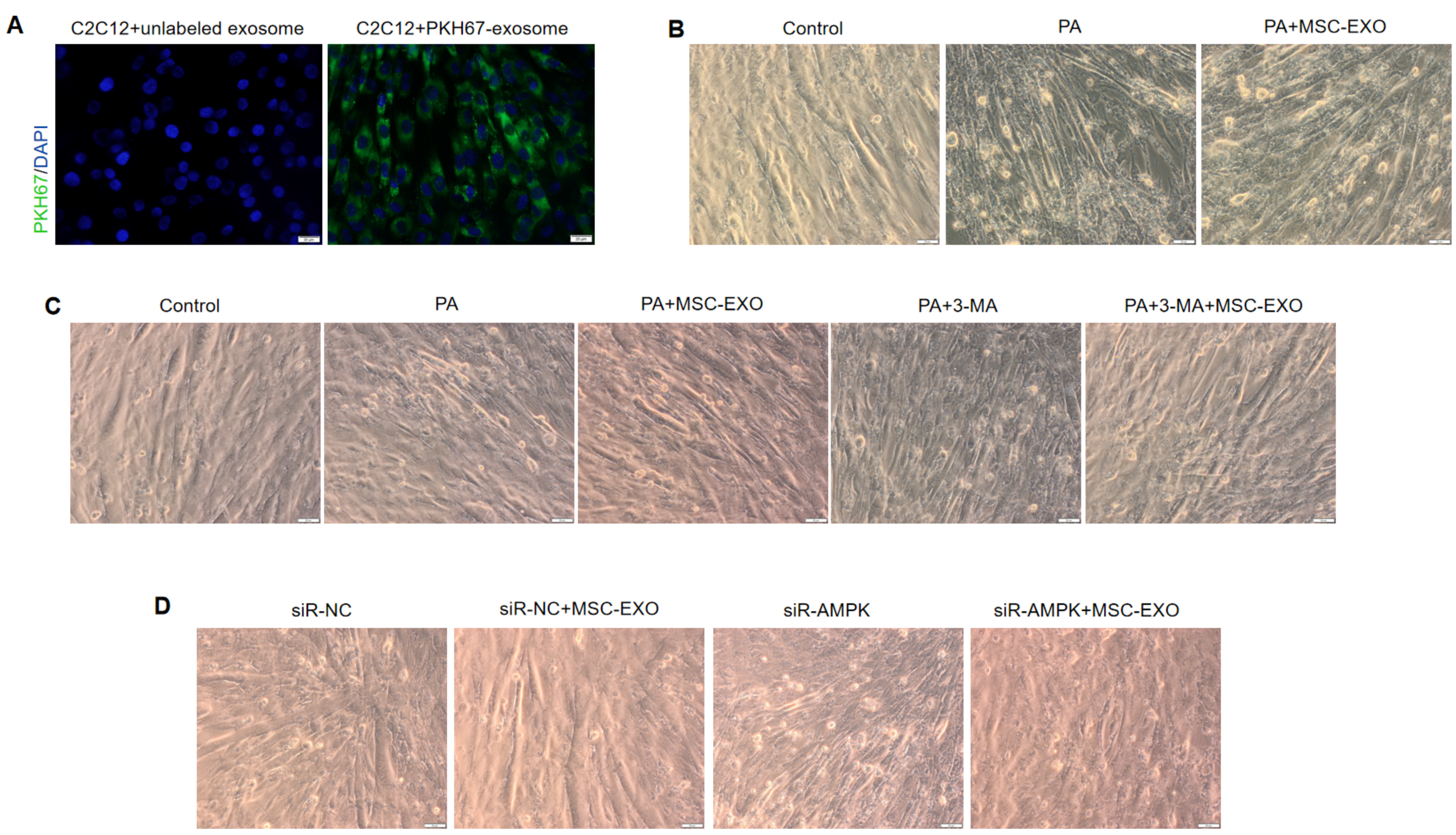

Supplement: Supplementary file 1 — Figure S1. Identification of hucMSCs. (A) Flow cytometry analysis of hucMSCs markers CD105, CD73, CD34 and HLA‐DR. (B) Oil Red O staining for adipogenic differentiation ability of hucMSCs (Scale bar, 20 μm). (C) Alizarin Red S staining for osteogenic differentiation ability of hucMSCs (Scale bar, 20 μm). Figure S2. hucMSCs alleviate diabetes‐induced muscle atrophy. (A) Intraperitoneal glucose tolerance test (IPGTT) and area under the curve (AUC) of db/db mice after hucMSC injection (n = 5–6 mice). (B) Intraperitoneal insulin tolerance test (IPITT) and AUC of db/db mice after hucMSC injection (n = 5–6 mice). (C) Body weight (n = 5–6 mice). (D) Lean mass detected by Dual‐energy X‐ray absorptiometry (DXA) (n = 5–6 mice). (E) Tibialis anterior (TA) muscle weight (n = 5–6 mice). (F) Number of muscle fibres (n = 3 mice). (G) Masson staining and Sirius Red staining of TA muscles (Scale bar, 50 μm). Data are mean ± SEM. (*P < 0.05, **P < 0.01, ***P < 0.001 by 2‐sided unpaired student's t‐test). Figure S3. hucMSCs alleviate obesity‐induced muscle atrophy. (A) IPGTT and AUC of HFD mice after hucMSC injection (n = 5–6 mice). (B) IPITT and AUC of HFD mice after hucMSC injection (n = 5–6 mice). (C) Body weight (n = 5–6 mice). (D) Lean mass detected by DXA (n = 5–6 mice). (E) TA muscle weight (n = 5–6 mice). (F) Number of muscle fibres (n = 3 mice). (G) Masson staining and Sirius Red staining of TA muscles (Scale bar, 50 μm). Data are mean ± SEM. (*P < 0.05, **P < 0.01, ***P < 0.001 by 2‐sided unpaired student's t‐test). Figure S4. hucMSCs ameliorate immobilization (IM)‐induced muscle atrophy. (A) Lean mass detected by DXA (n = 5 mice). (B) TA muscle weight (n = 5 mice). (C) Number of muscle fibres (n = 3 mice). (D) Masson staining and Sirius Red staining of TA muscles (Scale bar, 50 μm). Data are mean ± SEM. (*P < 0.05, **P < 0.01, ***P < 0.001 by 2‐sided unpaired student's t‐test). Figure S5. hucMSCs rescue atrophy‐associated impairment of the AMPK/ULK1 signalling and autophagy [file JCSM-14-915-s001.pdf]
