# Supplementary material for: Mesenchymal stromal cells ameliorate diabetes‐induced muscle atrophy through exosomes by enhancing AMPK/ULK1‐mediated autophagy
Source: J Cachexia Sarcopenia Muscle. 2023 Jan 27;14(2):915–29. doi: 10.1002/jcsm.13177 (PMC10067482; doi:10.1002/jcsm.13177)
Supplement: Supplementary file 2 — Data S1. Supporting Information [file JCSM-14-915-s002.docx]

**Supplementary references**

[S1]Li TS,  Shi H,  Wang L,  Yan C. Effect of Bone Marrow Mesenchymal Stem Cells on Satellite Cell Proliferation and Apoptosis in Immobilization-Induced Muscle Atrophy in Rats. [Med Sci Monit.](https://www.ncbi.nlm.nih.gov/pmc/articles/PMC5132424/) 2016; 22: 4651–4660.

[S2]Schilling BK,  Schusterman MA,  Kim DY,  Repko AJ,  Klett KC,  Christ GJ,  et al. Adipose-derived stem cells delay muscle atrophy after peripheral nerve injury in the rodent model. Muscle Nerve. 2019 May;59(5):603-610.

[S3]Artioli GG,  De Oliveira Silvestre JG,  Guilherme JP,  Baptista IL,  Ramos GV,  Da Silva WJ, et al. Embryonic stem cells improve skeletal muscle recovery after extreme atrophy in mice. Muscle Nerve. 2015 Mar;51(3):346-52.

[S4]Harrell CR, Jovicic N, Djonov V, Arsenijevic N, Volarevic V. Mesenchymal stem cell-derived exosomes and other extracellular vesicles as new remedies in the therapy of inflammatory diseases. Cells. 2019;8:1605.

[S5]Liu HW and Chang SJ. db/db Moderate Exercise Suppresses NF-κB Signaling and Activates the SIRT1-AMPK-PGC1α Axis to Attenuate Muscle Loss in Diabetic Mice. [Front Physiol.](https://www.ncbi.nlm.nih.gov/pmc/articles/PMC5987703/) 2018; 9: 636.

[S6]Aihara M,  Hirose N,  Katsuta W,  Saito F,  Maruyama H,  Hagiwara H. A new model of skeletal muscle atrophy induced by immobilization using a hook-and-loop fastener in mice. J Phys Ther Sci. 2017; 29(10).

[S7]Lee SM,  Lee SH,  Jung Y,  Lee Y,  Yoon JH,  Choi JY, et al. FABP3-mediated membrane lipid saturation alters fluidity and induces ER stress in skeletal muscle with aging. [Nat Commun.](https://www.ncbi.nlm.nih.gov/pmc/articles/PMC7653047/) 2020; 11: 5661.

[S8]Lu F,  Lu B,  Zhang L,  Wen J,  Wang M,  Zhang S, et al. Hydrogen sulphide ameliorating skeletal muscle atrophy in db/db mice via Muscle RING finger 1 S-sulfhydration. [J Cell Mol Med.](https://www.ncbi.nlm.nih.gov/pmc/articles/PMC7417732/) 2020; 24(16): 9362–9377.

[S9]Wang QL,  Zhuang X,  Sriwastva MK,  Mu J,  Teng Y,  Deng Z, et al. Blood exosomes regulate the tissue distribution of grapefruit-derived nanovector via CD36 and IGFR1 pathways. [Theranostics.](https://www.ncbi.nlm.nih.gov/pmc/articles/PMC6217058/) 2018; 8(18): 4912–4924.

[S10]Wang L,  Chopp M,  Szalad A,  Lu X,  Zhang Y,  Wang X, et al. Exosomes Derived From Schwann Cells Ameliorate Peripheral Neuropathy in Type 2 Diabetic Mice. [Diabetes.](https://www.ncbi.nlm.nih.gov/pmc/articles/PMC7085247/) 2020 Apr; 69(4): 749–759.

[S11][Zhao](https://pubmed.ncbi.nlm.nih.gov/?term=Zhao+B&cauthor_id=34167333) B, [Zhang](https://pubmed.ncbi.nlm.nih.gov/?term=Zhang+X&cauthor_id=34167333) XL, [Zhang](https://pubmed.ncbi.nlm.nih.gov/?term=Zhang+Y&cauthor_id=34167333) YL, [Lu](https://pubmed.ncbi.nlm.nih.gov/?term=Lu+Y&cauthor_id=34167333) YJ, [Zhang](https://pubmed.ncbi.nlm.nih.gov/?term=Zhang+W&cauthor_id=34167333) WT, [Lu](https://pubmed.ncbi.nlm.nih.gov/?term=Lu+S&cauthor_id=34167333) ST, et al. Human Exosomes Accelerate Cutaneous Wound Healing by Promoting Collagen Synthesis in a Diabetic Mouse Model. Stem Cells Dev. 2021 Sep 15;30(18):922-933.

[S12]Zhang C,  Mok J,  Seong Y,  Lau HC,  Kim D,  Yoon J, et al. PROKR1 delivery by cell-derived vesicles restores the myogenic potential of Prokr1-deficient C2C12 myoblasts. Nanomedicine. 2021;37:102448.

[S13]Xu Y,  Tan M,  Ma X,  Li H,  He X,  Chen Z,  et al. Human mesenchymal stem cells-derived conditioned medium inhibits hypoxia-induced death of neonatal porcine islets by inducing autophagy. [Xenotransplantation.](https://www.ncbi.nlm.nih.gov/pubmed/31578787) 2020;27(1):e12556.

[S14]Ebrahim N,  Ahmed IA,  Hussien NI,  Dessouky AA,  Farid AS,  Elshazly AM, et al. Mesenchymal Stem Cell-Derived Exosomes Ameliorated Diabetic Nephropathy by Autophagy Induction through the mTOR Signaling Pathway. [Cells.](https://www.ncbi.nlm.nih.gov/pubmed/30467302) 2018;7(12):226.

[S15]Guo Y, Yu W, Sun D, [Wang](https://pubmed.ncbi.nlm.nih.gov/?term=Wang+J&cauthor_id=24874076) J, [Li](https://pubmed.ncbi.nlm.nih.gov/?term=Li+C&cauthor_id=24874076) C, [Zhang](https://pubmed.ncbi.nlm.nih.gov/?term=Zhang+R&cauthor_id=24874076) R, et al. A novel protective mechanism for mitochondrial aldehyde dehydrogenase (ALDH2) in type 1 diabetes-induced cardiac dysfunction: role of AMPK-regulated autophagy. Biochim Biophys Acta. 2015 Feb;1852(2):319-31.

[S16]Wirth M, Joachim J, Tooze SA. Autophagosome formation–the role of ULK1 and Beclin1-PI3KC3 complexes in setting the stage. Semin Cancer Biol. 2013 Oct;23(5):301-9.

[S17]Egan DF, Shackelford DB, Mihaylova MM, Gelino S, Kohnz RA, Mair W, et al. Phosphorylation of ULK1 (hATG1) by AMP-activated protein kinase connects energy sensing to mitophagy. Science. 2011; 331:456–461.
